# iTRAQ-based proteomic analysis of myofibrillar contents and relevant synthesis and proteolytic proteins in soleus muscle of hibernating Daurian ground squirrels (*Spermophilus dauricus*)

**DOI:** 10.1186/s12953-016-0105-x

**Published:** 2016-11-08

**Authors:** Hui Chang, Shan-Feng Jiang, Kai Dang, Hui-Ping Wang, Shen-Hui Xu, Yun-Fang Gao

**Affiliations:** 1 Key Laboratory of Resource Biology and Biotechnology in Western China (College of Life Sciences, Northwest University), Ministry of Education, Xi’an, 710069 People’s Republic of China; 2 Shaanxi Key Laboratory for Animal Conservation, Northwest University, Xi’an, 710069 People’s Republic of China

**Keywords:** Daurian ground squirrel, Proteomic, Hibernation, Disuse atrophy, Myofibrillar protein, Synthesis and proteolysis

## Abstract

**Background:**

Daurian ground squirrels (*Spermophilus dauricus*) deviate from significant increase of protein catabolism and loss of myofibrillar contents during long period of hibernation inactivity.

**Methods:**

Here we use iTRAQ based quantitative analysis to examine proteomic changes in the soleus of squirrels in pre-hibernation, hibernation and post-hibernation states. The total proteolysis rate of soleus was measured by the release of the essential amino acid tyrosine from isolated muscles. Immunofluorescent analysis was used to determine muscle fiber cross-sectional area. Western blot was used for the validation of the quantitative proteomic analysis.

**Results:**

The proteomic responses to hibernation had a 0.4- to 0.8-fold decrease in the myofibrillar contractile protein levels of myosin-3, myosin-13 and actin, but a 2.1-fold increase in myosin-2 compared to pre-hibernation group. Regulatory proteins such as troponin C and tropomodulin-1 were 1.4-fold up-regulated and 0.7-fold down-regulated, respectively, in hibernation compared to pre-hibernation group. Moreover, 10 proteins with proteolytic function in hibernation, which was less than 14 proteins in the post-hibernation group, were up-regulated relative to the pre-hibernation group. The total proteolysis rates of soleus in hibernation and post-hibernation groups were significantly inhibited as compared with pre-hibernation group.

**Conclusion:**

These findings suggest that the myofibrillar remodeling and partial suppression of myofibrillar proteolysis were likely responsible for preventing skeletal muscle atrophy during prolonged disuse in hibernation. This is the first study where the myofibrillar contents and relevant synthesis and proteolytic proteins in slow soleus was discussed based on proteomic investigation performed on wild Daurian ground squirrels. Our results lay the foundation for further research in preventing disuse-induced skeletal muscle atrophy in mammals.

**Electronic supplementary material:**

The online version of this article (doi:10.1186/s12953-016-0105-x) contains supplementary material, which is available to authorized users.

## Background

In mammals, skeletal muscle accounts for more than 40 % of the mass of a given individual and provides critical functions in metabolism, energy expenditure, physical strength, and locomotor activity [1]. The skeletal muscle atrophy in response to disuse occurs during bed rest or spaceflight associated with the loss of muscle mass and the decline in muscle strength and power [2], muscular activity and cross-sectional area of muscle fiber [3]. The soleus muscle (SOL), which is predominantly composed of slow twitch fibers, is a postural muscle and more sensitive to disuse than fast-twitch muscles (extensor digitorum longus) and hybrid muscles (gastrocnemius) [4–6]. Disuse atrophy results in reduced protein content and a net loss of contractile proteins [7]. A proteomic study on rat soleus muscle after 3-week hindlimb unloading indicates that proteomic responses to disuse had a 0.2- to 0.6-fold decrease in the protein levels of myosin light chain 1 (MLC1), α-actin, tropomyosin β-chain, and troponins T [8]. Moreover, a number of results obtained showed that atrophic changes during a space flight or under head-down bed-rest are accompanied by decrease of total muscle protein [9] and myofibril proteins degradation [10]. Accordingly, disuse atrophy is supposed to be the result of shift of protein synthesis/proteolysis balance towards protein degradation increase [11], although many details remain unknown.

However, the skeletal muscle of hibernators appears to deviate from significant atrophy even after experiencing from extended disuse over three to four months, even six months in the cold North. It has been demonstrated that the muscle-fiber number and cross-sectional area were unchanged in gastrocnemius and biceps femoris of hibernating black bears (*Ursus americanus*), while protein concentration decreased in both muscles during the hibernation period, suggesting only marginal muscle atrophy [12]. In addition, it has also been reported that hibernating ground squirrels (*Citellus undulatus* and *Spermophilus dauricus*) have an evolutionarily determined adaptive mechanism of preservation or increase of slow fibers ratio [13], as the most economic and energetically advantageous, with proteins typical of them, whereas hindlimb unloading of non-hibernators (such as mouse) leads to activation of proteolysis and destruction of myofibrillar integrity, which contributes to considerable atrophy of soleus fibers [14]. Our previous research showed that SOL muscle mass to total body mass ratios (mg/g) were significantly higher in hibernating Daurian ground squirrels compared with that of rats after 14 days of hindlimb suspension, mirroring an effect protecting against disuse atrophy [15]. Daurian ground squirrels (*Spermophilus dauricus*) are obligatory hibernating mammals. They are found across a wide range of latitudes, from steppe and semi-desert and other arid regions of northern China. Hibernation of Daurian ground squirrels provides a useful model to study mechanisms that increases skeletal muscle resilience against atrophy and dysfunction after extended periods of disuse [16].

The study on measuring skeletal muscle protein metabolism of bears suggests that protein synthesis and breakdown are both lower in winter compared to summer but are equal during both early and late hibernation periods, indicating that bears are in protein balance during hibernation [17]. Which plays a predominant role in the maintenance of skeletal muscle homeostasis involved in the mechanism of protecting from muscle atrophy during prolonged disuse in hibernation, the increase of protein synthesis or the decrease of protein degradation? It is noteworthy that the protein biosynthesis category by overexpressed genes exhibits a highly significant enrichment in skeletal muscle of hibernating black bears (*Ursus americanus*) [18]. However, serum- and glucocorticoid-inducible kinase 1 (SGK1) can regulate muscle mass maintenance via downregulation of proteolysis and autophagy during hibernation in 13-lined ground squirrels (*Ictidomys tridecemlineatus*) [19], which is consistent with our previous report demonstrating that the inhibition of calpain activity and consequently calpain-mediated protein degradation by highly elevated calpastatin protein expression levels may be an important mechanism for preventing muscle protein loss during hibernation [15]. Recently, our group reported that the stable expression of atrogin-1 and MuRF1 may facilitate to prevent SOL [20] and extensor digitorum longus [21] muscle atrophy during hibernation. Although more and more regulatory factors involved in the protein metabolism of skeletal muscle during hibernation were found, the detailed mechanisms of protein synthesis and breakdown in hibernation are far from being elucidated.

To our knowledge we yet understand little about myofibrillar contents and relevant synthesis and proteolytic proteins in soleus muscle of hibernating ground squirrels. It is likely that novel mechanisms are involved but are not yet identified. Proteomics approaches are effective at identifying new protein signaling networks. Herein, we conducted isobaric tags for relative and absolute quantitation (iTRAQ) proteomics experiments in order to discover the hibernation-specific skeletal muscle proteomic changes. The aims of the present study were (1) to identify differentially expressed proteins among pre-hibernation, 60-d-hibernation and post-hibernation Daurian ground squirrels; (2) to explore the myofibrillar protein metabolism mechanism underlying the observed anti-atrophy effects in SOL of hibernating ground squirrels with the special ecological environment physiological adaptation.

## Methods

### Acquisition of animals

Acquisition and use of animals were approved by the Laboratory Animal Care Committee of the China Ministry of Health. As described previously by our laboratory [15], nine male Daurian ground squirrels were obtained from the Weinan region in the Shaanxi province of China and kept in the laboratory for three to four months after collection for acclimation purposes before they were split into groups. The animal colony room was maintained at a temperature range of 18–20 °C, and lighting was changed daily to coincide with local sunrise and sunset. Animals were given wood chips. Squirrels were provided with water and rodent food blocks, and supplemented with fresh fruit and vegetables. In November, three groups were placed in a cold room hibernaculum at (4–6 °C) (2 L: 22D dark). The dates of entering torpor were determined by putting sawdust on the back of each animal. Daily observations were made during the experimental period. Animals were matched for body mass and were randomly assigned to 3 groups: pre-hibernation: Control (no hibernation) animals investigated in late-autumn, about 30–40 d before hibernation; 60-d hibernation: Animals after two months of hibernation; post-hibernation: Animals two days after arousal from 112 ± 14 days of hibernation, with the SOL muscle collected 48 h after arousal. We used 3 animals per time point, then the 3 samples combined before iTRAQ labeling. In order to minimize the impact of individual differences and avoid interference from other factors, we choose animals with the same sex (male), the similar body (350 ± 20 g) weight and frequency of inter-bout arousal in hibernation for one sample. The SOL muscle was in a disuse state in the hibernation, but was not in a disuse state in the pre-hibernation and post-hibernation group.

### Immunofluorescent analysis

Ten-μm thick frozen muscle cross-sections were cut from the mid-belly of muscle at −20 °C with a cryostat (Leica, Wetzlar, CM1850, Germany), and stored at −80°Cfor further staining. Immunofluorescent analysis was used to determine muscle fiber cross-sectional area. After fixing in 4 % paraformaldehyde for 30 min, sections were permeabilized in 0.1 % Triton X-100/PBS for 30 min, blocked with 1 % bovine serum albumin (BSA) in PBS for 60 min at room temperature, and then incubated with the anti-laminin rabbit polyclonal antibody solution (1:50; Santa Cruz, CA, USA) at 4 °C overnight. The slides were rinsed twice in PBS and incubated with TRITC-labeled goat anti-rabbit IgG for 60 min and also counterstained with DAPI (0.5 μg/ml) for 30 min. Images were visualized using a confocal laser scanning microscope (Olympus, Osaka, Japan) at an objective magnification of 40× and were counted on at least 3 different fields or 600 cells of each sample.

### Muscle collection and protein preparation

All animal procedures were approved by the Northwest University Ethics Committee. Unless otherwise indicated, all chemicals were purchased from Sigma-Aldrich. Animals were anesthetized with 90 mg/kg sodium pentobarbital i.p. After SOL muscles in ground squirrels were excised from both legs, body mass and wet mass of SOL were recorded. At the end of surgical intervention, the animals were sacrificed by an overdose injection of sodium pentobarbital. Then the SOL muscle samples were ground into powder in liquid nitrogen, extracted with Lysis buffer (7 M Urea, 2 M Thiourea, 4℅CHAPS, 40 mM Tris–HCl, pH 8.5) containing 1 mM PMSF and 2 mM EDTA. After 5 min, 10 mM DTT (final concentration) was added to the samples. The suspension was sonicated at 200 W for 15 min and then centrifuged at 4 °C, 30,000 g for 15 min. The supernatant was mixed well with 5× volume of chilled acetone containing 10 % (v/v) TCA and incubated at −20 °C overnight. After centrifugation at 4 °C, 30,000 g, the supernatant was discarded. The precipitate was washed with chilled acetone three times. The pellet was air-dried and dissolved in Lysis buffer (7 M urea, 2 M thiourea, 4 % NP40, 20 mM Tris–HCl, pH 8.0-8.5). The suspension was sonicated at 200 W for 15 min and centrifuged at 4 °C, 30,000 g for 15 min. The supernatant was transferred to another tube. To reduce disulfide bonds in proteins of the supernatant, 10 mM DTT (final concentration) was added and incubated at 56 °C for 1 h. Subsequently, 55 mM IAM (final concentration) was added to block the cysteines, incubated for 1 h in the darkroom. The supernatant was mixed well with 5× volume of chilled acetone for 2 h at −20 °C to precipitate proteins. After centrifugation at 4 °C, 30,000 g, the supernatant was discarded, and the pellet was air-dried for 5 min, dissolved in 500 μL 0.5 M TEAB (Applied Biosystems, Milan, Italy), and sonicated at 200 W for 15 min. Finally, samples were centrifuged at 4 °C, 30,000 g for 15 min. The supernatant was transferred to a new tube and kept at −80 °C for further analysis.

### iTRAQ Labeling and SCX fractionation

iTRAQ analysis was implemented at Beijing Genomics Institute (BGI, Shenzhen, China). Total protein (100 μg) was taken out of each sample solution and then the protein was digested with Trypsin Gold (Promega, Madison, WI, USA) with the ratio of protein/trypsin (30/1) at 37 °C for 16 h. After trypsin digestion, peptides were dried by vacuum centrifugation. Peptides were reconstituted in 0.5 M TEAB and processed according to the manufacture’s protocol for 8-plex iTRAQ reagent (Applied Biosystems). Briefly, one unit of iTRAQ reagent was thawed and reconstituted in 24 μL isopropanol. SOL muscle samples were labeled with the iTRAQ tags as follow: pre-hibernation (tag 113), 60-day hibernation (tag 114) and post-hibernation (tag 116). The peptides were labeled with the isobaric tags, incubated at room temperature for 2 h. The labeled peptide mixtures were then pooled and dried by vacuum centrifugation.

SCX chromatography was performed with a LC-20AB HPLC Pump system (Shimadzu, Kyoto, Japan). The iTRAQ-labeled peptide mixtures were reconstituted with 4 mL buffer A (25 mM NaH_2_PO_4_ in 25 % ACN, pH 2.7) and loaded onto a 4.6 × 250 mm Ultremex SCX column containing 5-μm particles (Phenomenex). The peptides were eluted at a flow rate of 1 mL/min with a gradient of buffer A for 10 min, 5–60 % buffer B (25 mM NaH_2_PO_4_, 1 M KCl in 25 % ACN, pH 2.7) for 27 min, 60–100 % buffer B for 1 min. The system was then maintained at 100 % buffer B for 1 min before equilibrating with buffer A for 10 min prior to the next injection. Elution was monitored by measuring the absorbance at 214 nm, and fractions were collected every 1 min. The eluted peptides were pooled into 20 fractions, desalted with a Strata X C18 column (Phenomenex) and vacuum-dried.

### LC-ESI-MS/MS analysis based on Triple TOF 5600

Each fraction was resuspended in buffer A (5 % ACN, 0.1 % FA) and centrifuged at 20,000 g for 10 min, the final concentration of peptide was about 0.5 μg/μL on average. 10 μL supernatant was loaded on a LC-20 AD nano HPLC(Shimadzu, Kyoto, Japan) by the autosampler onto a 2 cm C18 trap column. Then, the peptides were eluted onto a 10 cm analytical C18 column (inner diameter 75 μm) packed in-house. The samples were loaded at 8 μL/min for 4 min, then the 35 min gradient was run at 300 nL/min starting from 2 to 35 % buffer B (95 % ACN, 0.1 % FA), followed by 5 min linear gradient to 60 %, then followed by 2 min linear gradient to 80 %, and maintenance at 80 % buffer B for 4 min, and finally return to 5 % in 1 min. Data acquisition was performed with a Triple TOF 5600 System (AB SCIEX, Concord, ON) fitted with a Nanospray IIIsource (AB SCIEX, Concord, ON) and a pulled quartz tip as the emitter (New Objectives, Woburn, MA). Data was acquired using an ion spray voltage of 2.5 kV, curtain gas of 30 psi, nebulizer gas of 15 psi, and an interface heater temperature of 150 °C. The MS was operated with a RP of greater than or equal to 30,000 FWHM for TOF MS scans. For IDA, survey scans were acquired in 250 ms and as many as 30 product ion scans were collected if exceeding a threshold of 120 counts per second (counts/s) and with a 2+ to 5+ charge-state. Total cycle time was fixed to 3.3 s. Q2 transmission window was 100 Da for 100 %. Four time bins were summed for each scan at a pulser frequency value of 11 kHz through monitoring of the 40 GHz multichannel TDC detector with four-anode channel detect ion. A sweeping collision energy setting of 35 ± 5 eV coupled with iTRAQ adjust rolling collision energy was applied to all precursor ions for collision induced dissociation. Dynamic exclusion was set for 1/2 of peak width (15 s), and then the precursor was refreshed off the exclusion list.

### Data analysis

Raw data files acquired from the Orbitrap were converted into MGF files using Proteome Discoverer 1.2 (PD 1.2, Thermo), [5600 msconverter] and the MGF file were searched. Proteins identification was performed by using Mascot search engine (Matrix Science, London, UK, version 2.3.02) against database containing 28,942 sequences (up to date 2014-3-11). For protein identification, a mass tolerance of 0.05 Da (ppm) was permitted for intact peptide masses and 0.1 Da for fragmented ions, with allowance for one missed cleavages in the trypsin digests. Gln- > pyro-Glu (N-term Q), Oxidation (M), Deamidated (NQ) as the potential variable modifications, and Carbamidomethyl (C), iTRAQ8plex (N-term), iTRAQ8plex (K) as fixed modifications. The charge states of peptides were set to +2 and +3. Specifically, an automatic decoy data base search was performed in Mascot by choosing the decoy checkbox in which a random sequence of database is generated and tested for raw spectra as well as the real database. To reduce the probability of false peptide identification, only peptides with significance scores (≥20) at the 99 % confidence interval by a Mascot probability analysis greater than“identity” were counted as identified. And each confident protein identification involves at least one unique peptide. For protein quantitation, it was required that a protein contains at least two unique peptides. The quantitative protein ratios were weighted and normalized by the median ratio in Mascot. We only used ratios with p-values < 0.05, and only fold changes of >1.2 was considered as significant.

### Function method description

Functional annotations of the proteins were conducted using Blast2GO program against the non-redundant protein database (NR; NCBI). The KEGG database (http://www.genome.jp/kegg/) and the COG database (http://www.ncbi.nlm.nih.gov/COG/) were used to classify and group these identified proteins.

### Pathway analysis

KEGG PATHWAY is a database resource and a collection of manually drawn pathway maps [22, 23] representing our knowledge on the molecular interaction and reaction networks between the identified differentially expressed proteins in 60-d hibernation and post-hibernation ground squirrels groups compared to the pre-hibernation groups. Molecules are represented as nodes, and the biological relationship between two nodesis represented as an edge (line).

### Western blotting

To confirm the reliability of iTRAQ based quantitative analysis, protein samples used for iTRAQ were further examined by western blot which were undertaken as previously described [24]. Briefly, total protein was extracted from the SOL muscle of ground squirrels and solubilized in a sample buffer (100 mM Tris, pH 6.8, 5 % 2-β-mercaptoethanol, 5 % glycerol, 4 % SDS, and bromophenol blue), with muscle protein extracts resolved by SDS-PAGE using Laemmli gels (10 % gel with an acrylamide/bisacrylamide ratio of 37.5 : 1 for EEF-2; and 12 % gel with an acrylamide/bisacrylamide ratio of 29 : 1 for RPS8, proteasome 20S α5, CAPNS1, actin, and troponin C. After electrophoresis, the proteins were electrically transferred to PVDF membranes (0.45 μm pore size) using a Bio-Rad semi-dry transfer apparatus. The blotted membranes were blocked with 1 % BSA in Tris-buffered saline (TBS; 150 mM NaCl, 50 mM Tris–HCl, pH 7.5) and incubated with rabbit anti-Proteasome 20S α5, rabbit anti-Actin, rabbit anti-Troponin C, rabbit anti-EEF2 and rabbit anti-RPS8 (1:1000, Abcam, Cambridge, MA, USA) and rabbit anti-CAPNS1 (1:1000, Sigma, St. Louis, MO, USA) in TBS containing 0.1 % BSA at 4 °C overnight. After washing 3 times, the membranes were then incubated with HRP-conjugated anti-rabbit secondary antibodies (Pierce Chemical Co., Dallas, USA) for 1 h at room temperature. After another washing, immunoblots were visualized using enhanced chemiluminescence (ECL) reagents (Thermo, Rockford, USA.) according to the manufacturer’s protocols. Quantification analysis of blots was performed with the NIH Image J software.

### Total protein breakdown in incubated muscles

Rates of protein degradation was determined by the release of tyrosine in incubated soleus muscles as previously described [25]. As tyrosine is present in all proteins, its release reflects total protein breakdown. Briefly, the ground squirrels were anesthetized and the soleus muscles were gently dissected and excised with intact tendons. Immediately after weighing, muscles were secured with custom plastic clips at approximately resting length in order to better maintain their energy level and protein balance. Muscles were incubated for 30 min in oxygenated (O_2_ : CO_2_ = 95 : 5) Krebs-Henseleit buffer (pH 7.4) containing 5 mM glucose and 0.15 mM pyruvate. After preincubation, one muscle was homogenized in 3 % perchloric acid for determination of tissue levels of free tyrosine. The contralateral muscle was then blotted and transferred to a new incubation well containing 3 mL Krebs-Henseleit/glucose/pyruvate buffer and 0.5 mM cycloheximide, which inhibited reincorporation of amino acids by protein synthesis. Three hours after incubation, muscles were removed, blotted dry, and frozen in liquid nitrogen. Then the muscle and medium concentrations of free tyrosine were measured by high performance liquid chromatography (Agilent HPLC system, Column: Agilent TC-C18, 5 μm, 4.6 × 250 mm, USA), which was equipped with a binary pump (Agilent G1312A) and a fluorescence detector (Agilent G1321A). Rates of protein degradation are given as nmol tyrosine per gram of muscle wet weight per 3 h.

### Statistical analyses

A one-way ANOVA with Fisher’s LSD post hoc test was used to determine group differences, and the ANOVA–Dunnett’s T3 method was used when no homogeneity was detected. SPSS 19.0 was used for all statistical tests. Statistical significance was accepted for all tests at *P* < 0.05.

## Results

### Body weight, soleus muscle wet weight and muscle fiber cross-sectional area (CSA)

**Fig. 1:**
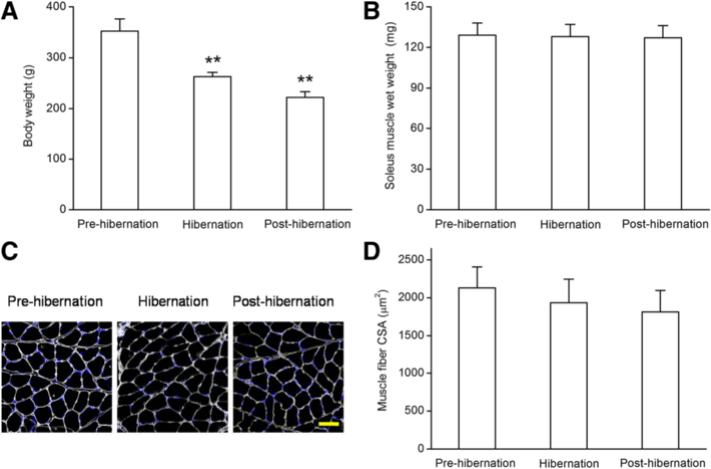
Body weight (**a**) and soleus muscle wet weight (**b**) of Daurian ground squirrels in pre-hibernation, 60-d and post-hibernation groups. Representative images (**c**) and bar graph (**d**) showing the changes in cross-sectional area of soleus muscle from 3 groups. White represents the anti-laminin stain of interstitial tissue counterstained with DAPI (*blue*) for nuclei identifcation. Scale bar = 50 μm. Values are means ± SEM, *n* = 6 in each group. **P < 0.01 versus pre-hibernation group

There was a steady decline in mean body weight from 352 ± 24 g in pre-hibernation to 263 ± 8 g after 60 days of hibernation, and there was even a sharp body weight decrease to 222 ± 11 g in post-hibernation. In other words, the ground squirrels lost 25 and 40 % of the body weight after hibernation for 60 days and post-hibernation, respectively (Fig. 1a). However, the SOL muscle wet weights were decreased slightly in 60-d or post-hibernation ground squirrels in comparison with the pre-hibernation group (Fig. 1b). Meanwhile, the CSA of SOL muscle also showed no significant decrease in 60-d or post-hibernation groups as compared with the pre-hibernation group (Fig. 1c and d).

### Proteomics analysis

In the present study, an iTRAQ-based quantitative proteomics approach in combination with LC-ESI-MS/MS was applied to investigate differentially expressed proteins in the SOL of pre-hibernation, 60-d hibernation and post-hibernation ground squirrels. Proteomics analysis identified 11,897 peptides mapped to 2059 proteins. iTRAQ ratio of > 1.20 and < 0.83 (*P*-value < 0.05) was used to define proteins that are significantly up-regulated or down-regulated, respectively. With this filter, we identified 170 and 333 differentially regulated proteins in the 60-d and post-hibernation groups relative to the pre-hibernation ground squirrels, respectively. Besides, 273 differentially regulated proteins were identified in the post-hibernation group compared to the 60-d hibernation ground squirrels. These proteins were subjected to gene-ontology enrichment. Among the three groups, the differences between 60-d hibernation and pre-hibernation were smaller (96 vs 248 up-regulated proteins and 74 vs 85 down-regulated proteins) as compared with the differences between post-hibernation and pre-hibernation ground squirrels. Moreover, 216 up-regulated proteins and 57 down-regulated were identified in the post-hibernation group compared to the 60-d hibernation ground squirrels. Specifically, there were 248 up-regulated expressed proteins between post-hibernation and pre-hibernation ground squirrels, almost one third of the total number (776) of differentially expressed proteins among three ground squirrels groups.

### Gene ontology (GO) classification of differentially expressed proteins

**Fig. 2:**
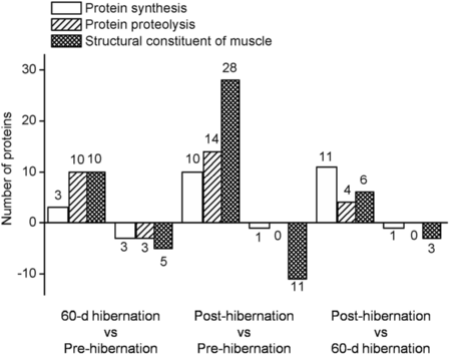
GO classification of differentially expressed proteins

To elucidate the biological significance of the 776 differentially modified proteins, we performed GO analysis and categorized these proteins according to their molecular function and biological process using the KEGG database (http://www.genome.jp/kegg/) and the COG database (http://www.ncbi.nlm.nih.gov/COG/). Of the 776 proteins, 123 were selected from these analyses and separated into 3 categories according to their molecular function: protein synthesis, protein proteolysis and structural constituent of muscle. Figure 2 showed the number of significantly (*P* < 0.05) differentially expressed proteins in the 3 categories. Proteins were annotated according to the gene ontology (GO) classification (Tables 1, 2 and 3).

**Table 1:** Summary table showing significantly up-regulated or down-regulated proteins in SOL of Daurian ground squirrels identified by iTRAQ

Acc no. (NCBI)	Prot name [Ictidomys tridecemlineatus]	MW (Da)	Peptide	Score	60-d hibernation vs pre-hibernation		Post-hibernation vs pre-hibernation		Post-hibernation vs 60-hibernation	
					Fold change	Sig	Fold change	Sig	Fold change	Sig
Up-regulated proteins										
gi|532099039	elongation factor 1-beta isoform *X*2	32189	6	175	1.348	*	1.202	*		
gi|532060958	60S ribosomal protein L39-like	9445	1	58	1.791	*				
gi|532064086	polyadenylate-binding protein 4 isoform X1	87231	7	188	1.508	*				
gi|532076481	ATP-dependent RNA helicase DDX3X	60127	11	481			1.283	*		
gi|532064280	40S ribosomal protein S8	34224	6	177			1.349	*		
gi|532090722	eukaryotic initiation factor 4A-II isoform X1	51896	9	769			1.232	*		
gi|532100826	60S ribosomal protein L38 isoform *X*2	13441	2	71			1.679	*		
gi|532073691	60S ribosomal protein L30-like	17542	2	66			1.366	*		
gi|532092237	60S ribosomal protein L10-like isoform X1	32972	1	68			1.276	*	1.419	*
gi|532092821	elongation factor 2	116340	23	1556			1.227	*		
gi|532114042	60S acidic ribosomal protein P2 isoform X3	15038	5	699			1.455	*		
gi|532060529	elongation factor 1-delta isoform X4	37312	8	983			1.476	*	1.292	*
gi|532064519	60S ribosomal protein L11	13027	1	53					1.329	*
gi|532085355	40S ribosomal protein S25	22308	2	114					1.228	*
gi|532098257	60S ribosomal protein L22	14812	2	160					1.364	*
gi|532071999	60S ribosomal protein L23	19837	5	246					1.345	*
gi|532082343	60S ribosomal protein L27a	27522	2	86					1.214	*
gi|532110684	40S ribosomal protein S4, X isoform	38325	3	192					1.244	*
gi|532082140	40S ribosomal protein S13	22992	4	72					1.238	*
gi|532094470	60S ribosomal protein L26	24853	1	34					1.225	*
gi|532075928	40S ribosomal protein S15a	18594	3	117					1.223	*
Down-regulated proteins										
gi|532085355	40S ribosomal protein S25	22308	2	114	0.758	*				
gi|532098257	60S ribosomal protein L22	14812	2	160	0.723	*				
gi|532083400	40S ribosomal protein S7	33087	3	90	0.735	*				
gi|532066199	60S ribosomal protein L10a	25228	2	105			0.74	*		
gi|532096351	28S ribosomal protein S25	24779	1	51					0.588	*

Acc no. (Accession number), Prot name (protein name), MW (Molecular mass), Peptide (number of peptides matched), Score (Mowse score), Sig (*P-value < 0.05 was considered statistically significant)

**Table 2:** Summary table showing significantly up-regulated or down-regulated proteins with protein proteolysis function in SOL of Daurian ground squirrels identified by iTRAQ

Acc no. (NCBI)	Prot name [Ictidomys tridecemlineatus]	MW (Da)	Peptide	Score	60-d hibernation vs pre-hibernation		Post-hibernation vs pre-hibernation		Post-hibernation vs 60-d hibernation	
					Fold change	Sig	Fold change	Sig	Fold change	Sig
Up-regulated proteins										
gi|532073955	proteasome subunit alpha type-6	28470	8	480	1.23	*	1.248	*		
gi|532105099	proteasome subunit alpha type-5	30824	7	614	1.226	*	1.226	*		
gi|59800155	26S protease regulatory subunit 10B	53861	7	589	1.281	*	1.264	*		
gi|532070252	26S proteasome non-ATPase regulatory subunit 5 isoform X1	63125	2	46	1.517	*				
gi|532091148	26S proteasome non-ATPase regulatory subunit 4	48541	5	133	1.488	*				
gi|532103645	proteasome subunit beta type-6 isoform X1	27252	3	115	1.295	*				
gi|532073989	proteasome subunit alpha type-3 isoform X1	34727	5	462	1.209	*				
gi|532066291	cytosol aminopeptidase	66612	11	731	1.202	*				
gi|532101732	26S protease regulatory subunit 6B isoform X1	56577	4	375	1.246	*				
gi|532088665	26S protease regulatory subunit 6A	60713	9	638	1.32	*				
gi|532080894	calpastatin	113441	7	179			1.226	*		
gi|532073188	endoplasmin	117277	14	399			1.316	*		
gi|532076563	ubiquitin-like modifier-activating enzyme 1 isoform *X*2	135211	19	1304			1.242	*		
gi|532069763	xaa-Pro aminopeptidase 1-like isoform X1	88255	5	128			1.485	*		
gi|532062055	proteasome subunit alpha type-4	37964	6	247			1.289	*		
gi|532114683	calpain small subunit 1	31666	4	257			1.301	*	1.488	*
gi|532105581	ubiquitin carboxyl-terminal hydrolase 5 isoform X1	112519	10	369			1.221	*		
gi|532082471	acylamino-acid-releasing enzyme	91596	7	160			1.567	*		
gi|532088974	prolyl endopeptidase	95929	11	396			1.385	*		
gi|532078165	ubiquitin carboxyl-terminal hydrolase isozyme L3	30900	4	212			1.591	*	1.552	*
gi|532097874	ubiquitin fusion degradation protein 1 homolog isoform X1	41760	5	206			1.419	*		
gi|532116708	proteasome subunit beta type-1	30778	7	506					1.283	*
gi|532085114	26S proteasome non-ATPase regulatory subunit 12 isoform X1	66713	4	67					1.457	*
Down-regulated proteins										
gi|532064840	ubiquinone biosynthesis protein COQ9, mitochondrial	38864	6	713	0.811	*				
gi|532078642	poly(rC)-binding protein 2 isoform X1	54651	5	470	0.732	*				
gi|532086828	cullin-5	114587	6	160	0.774	*				

Acc no. (Accession number), Prot name (protein name), MW (Molecular mass), Peptide (number of peptides matched), Score (Mowse score), Sig (*P-value < 0.05 was considered statistically significant)

**Table 3:** Summary table showing significantly up-regulated or down-regulated proteins of structural constituent of SOL muscle in Daurian ground squirrels identified by iTRAQ

Acc no. (NCBI)	Prot name [Ictidomys tridecemlineatus]	MW (Da)	Peptide	Score	60-d hibernation vs pre-hibernation		Post-hibernation vs pre-hibernation		Post-hibernation vs 60-d hibernation	
					Fold change	Sig	Fold change	Sig	Fold change	Sig
Up-regulated proteins										
gi|532098453	small muscular protein isoform *X*2	12381	3	504	1.238	*	1.392	*		
gi|532095619	cofilin-1	26628	7	639	1.239	*	1.535	*		
gi|532065022	tubulin polymerization-promoting protein family member 3	27600	2	147	1.607	*	1.481	*		
gi|532085001	myosin light chain 4 isoform *X*2	27241	6	968	1.434	*	1.579	*		
gi|532097561	myelin basic protein	44089	2	69	1.456	*				
gi|532069819	nebulin-related-anchoring protein	245551	22	744	1.211	*				
gi|532094579	myosin-2	244418	102	66551	2.135	*				
gi|532087098	PDZ and LIM domain protein 3 isoform X1	45822	12	3700	1.304	*				
gi|532062716	troponin C	22795	11	5693	1.358	*				
gi|532080240	vimentin	54132	26	3859	1.211	*			1.828	*
gi|532089364	myosin-binding protein H	60653	11	835			1.696	*		
gi|532081847	zyxin	72401	2	97			1.371	*		
gi|532111896	tubulin alpha-8 chain isoform X1	60805	15	2177			1.355	*		
gi|532063429	F-actin-capping protein subunit beta isoform *X*2	36610	11	763			1.219	*		
gi|532074211	nidogen-2 isoform X1	165553	7	164			1.467	*		
gi|532076843	myozenin-3	30065	6	801			1.401	*		
gi|532100104	myosin light polypeptide 6 isoform X1	20132	8	2096			1.576	*		
gi|532099239	moesin-like	87289	14	565			1.248	*		
gi|532103138	myosin regulatory light chain 12B	24691	6	374			1.771	*		
gi|532081115	talin-1	321070	21	790			1.288	*	1.302	*
gi|532088445	myosin regulatory light polypeptide 9 isoform X1	24375	5	323			1.68	*		
gi|532095126	PDZ and LIM domain protein 7	60050	6	361			1.653	*		
gi|532111349	ankyrin-1 isoform X1	242338	33	1740			2.69	*		
gi|532098546	tubulin beta-5 chain-like	46713	12	1245			1.367	*		
gi|532061621	annexin A2	49151	21	2021			1.502	*		
gi|532068053	myocilin	59868	4	159			2.837	*		
gi|532100974	neurofilament heavy polypeptide	158649	7	679			1.55	*		
gi|532110489	neurofilament medium polypeptide	123496	7	571			1.677	*		
gi|532089731	tubulin alpha-4A chain	56718	18	3110			1.396	*		
gi|532089733	tubulin alpha-1D chain-like	57019	18	3316			2.103	*		
gi|532063781	protein 4.1 isoform X1	120337	9	505			2.552	*		
gi|532111734	alpha-actinin-4 isoform X1	125310	18	2664			1.228	*		
gi|532072736	beta-adducin isoform X1	100809	3	84			1.759	*		
gi|532083652	tubulin beta-2A chain isoform X1	56576	21	2455			1.637	*	1.687	*
gi|532081219	tropomodulin-1	49085	4	141					1.509	*
gi|532102653	tubulin beta-4B chain isoform X1	55122	22	3594					1.25	*
gi|532062201	fibrillin-1	340347	14	616					1.685	*
Down-regulated proteins										
gi|532094519	myosin-3	287961	49	33292	0.587	*	0.399	*	0.758	*
gi|532085261	transgelin isoform *X*2	28099	11	691	0.784	*				
gi|532062201	fibrillin-1	340347	14	616	0.534	*				
gi|532072041	telethonin	21240	8	817	0.811	*				
gi|532081219	tropomodulin-1	49085	4	141	0.701	*				
gi|532103167	actin	48418	23	58752			0.823	*	0.806	*
gi|532059726	xin actin-binding repeat-containing protein 2-like	476702	6	84			0.774	*		
gi|532069819	nebulin-related-anchoring protein	245551	22	744			0.747	*		
gi|532094510	myosin-13	285781	37	26764			0.478	*		
gi|532077683	kelch-like protein 41	85237	21	1666			0.772	*		
gi|532097391	supervillin	300575	12	302			0.789	*		
gi|532112765	cytoplasmic dynein 1 heavy chain 1	620997	16	383			0.807	*		
gi|532113339	obscurin, partial	143269	19	783			0.829	*		
gi|532112836	flotillin-1	56752	3	161			0.806	*		
gi|532062845	filamin-B	333949	10	1568			0.743	*		
gi|532094579	myosin-2	244418	102	66551					0.482	*

Acc no. (Accession number), Prot name (protein name), MW (Molecular mass), Peptide (number of peptides matched), Score (Mowse score), Sig (*P-value < 0.05 was considered statistically significant)

### Pathway analysis

**Fig. 3:**
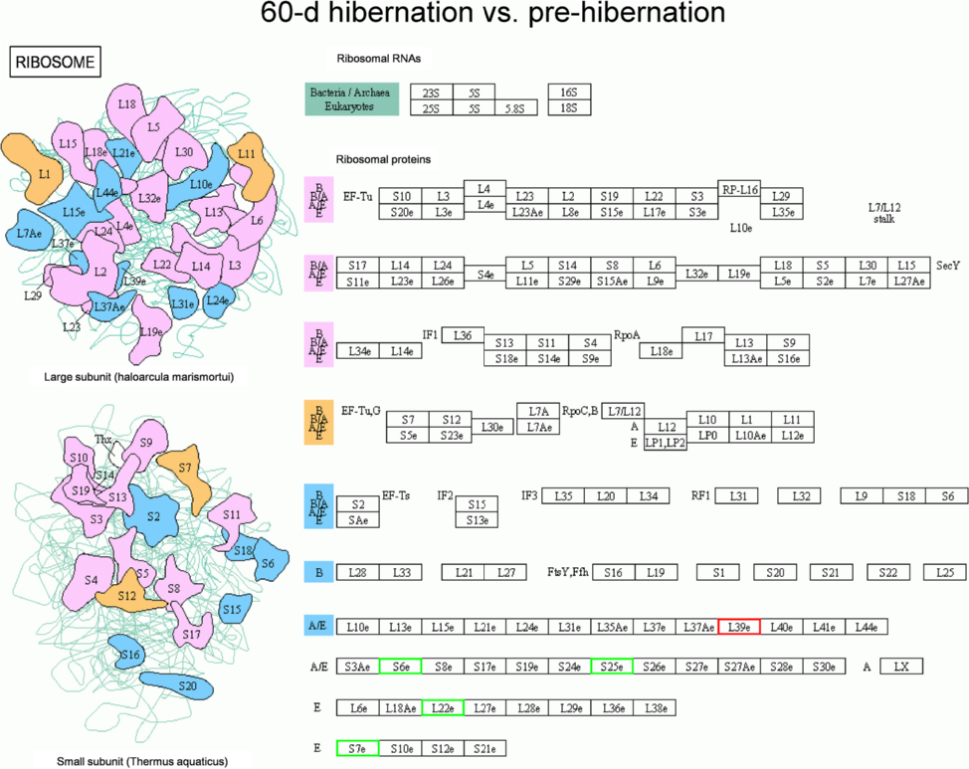
Pathway analysis using the Kyoto Encyclopedia of Genes and Genomes (KEGG) Pathway database. Differentially expressed proteins enriched in the ribosomal assembly pathway in the 60-d hibernation ground squirrels group compared to the pre-hibernation group. Red indicates up-regulated expressed proteins while green indicates down-regulated expressed proteins. L39e: gi|532060958, 60S ribosomal protein L39-like; S6e: gi|532100665, 40S ribosomal protein S6; S25e: gi|532085355, 40S ribosomal protein S25; L22e: gi|532098257, 60S ribosomal protein L22; S7e: gi|532083400, 40S ribosomal protein S7

**Fig. 4:**
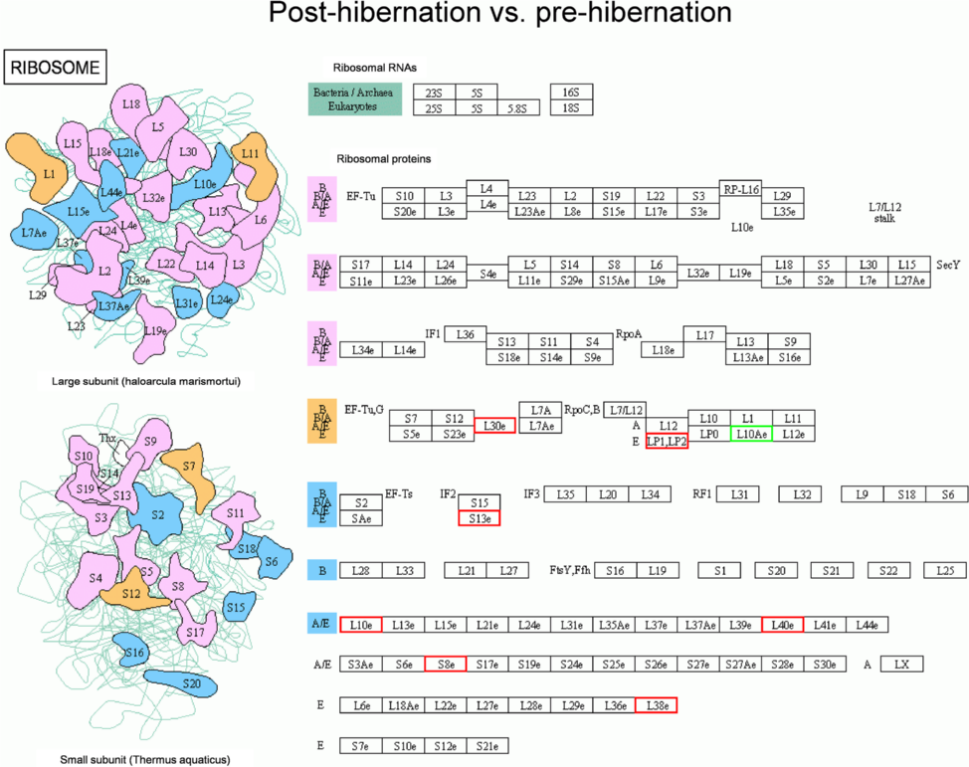
Pathway analysis using the KEGG Pathway database. Differentially expressed proteins enriched in the ribosomal assembly pathway in the post-hibernation ground squirrels group compared to the pre-hibernation group. Red indicates up-regulated expressed proteins while green indicates down-regulated expressed proteins. L30e: gi|532073691, 60S ribosomal protein L30-like; LP1, LP2: gi|532114042, 60S acidic ribosomal protein P2 isoform X3; L10Ae: gi|532066199, 60S ribosomal protein L10a; S13e: gi|532082140, 40S ribosomal protein S13; L10e: gi|532092237, 60S ribosomal protein L10-like isoform X1; L40e: gi|532068244, ubiquitin-60S ribosomal protein L40-like; S8e: gi|532064280, 40S ribosomal protein S8; L38e: gi|532100826, 60S ribosomal protein L38 isoform *X*2

**Fig. 5:**
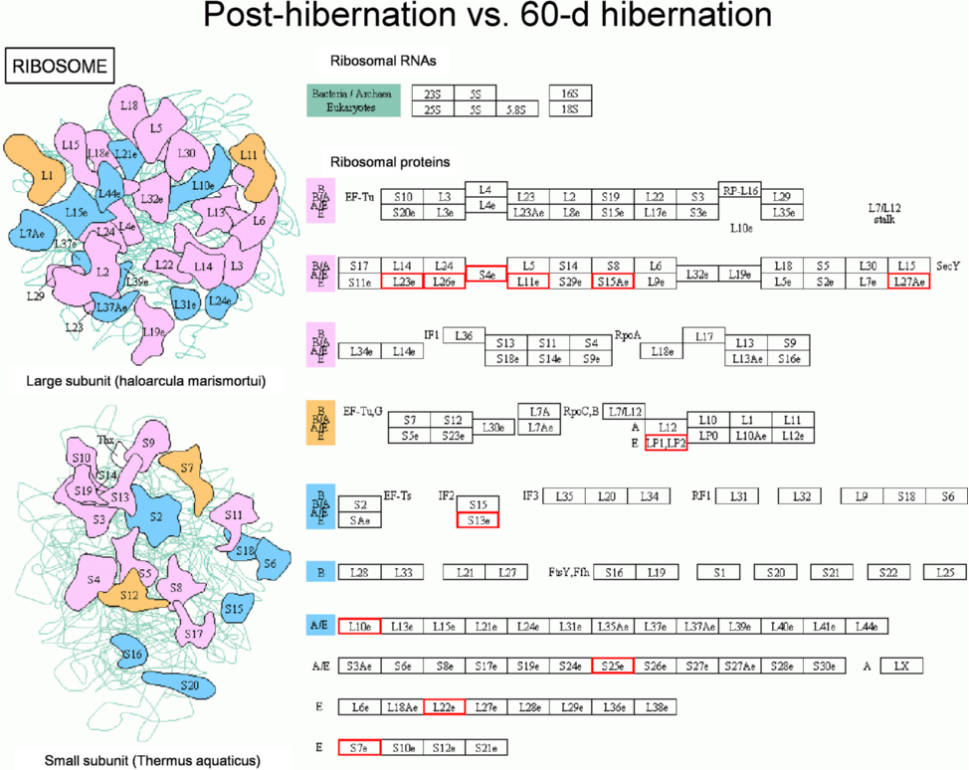
Pathway analysis using the KEGG Pathway database. Differentially expressed proteins enriched in the ribosomal assembly pathway in the post-hibernation ground squirrels group compared to the 60-d hibernation group. Red indicates up-regulated expressed proteins. L23e: gi|532071999, 60S ribosomal protein L23; L26e: gi|532094470, 60S ribosomal protein L26; S4e: gi|532110684, 40S ribosomal protein S4; L11e: gi|532064519, 60S ribosomal protein L11; S15Ae: gi|532075928, 40S ribosomal protein S15a; L27Ae: gi|532082343, 60S ribosomal protein L27a; LP1,LP2: gi|532114042, 60S acidic ribosomal protein P2 isoform X3; S13e: gi|532082140, 40S ribosomal protein S13; L10e: gi|532092237, 60S ribosomal protein L10-like isoform X1; S25e: gi|532085355, 40S ribosomal protein S25; L22e: gi|532098257, 60S ribosomal protein L22; S7e: gi|532083400, 40S ribosomal protein S7

Ribosomes are the sites of protein synthesis, the increased expression of these proteins may have improved protein synthesis in hibernating ground squirrels. Of the proteins identified by iTRAQ that were differentially expressed between 60-d hibernation group and pre-hibernation group, 5 enriched in the ribosomal assembly pathways (60S ribosomal protein L39-like, 40S ribosomal protein S6, 40S ribosomal protein S25, 60S ribosomal protein L22, 40S ribosomal protein S7, Fig. 3), and 8 of the proteins that were differentially expressed between post-hibernation group and pre-hibernation group enriched in this category (60S ribosomal protein L30-like, 60S acidic ribosomal protein P2 isoform X3, 60S ribosomal protein L10a, 40S ribosomal protein S13, 60S ribosomal protein L10-like isoform X1, ubiquitin-60S ribosomal protein L40-like, 40S ribosomal protein S8, 60S ribosomal protein L38 isoform *X*2, Fig. 4). Meanwhile, 12 up-regulated proteins were enriched in the ribosomal assembly pathways between post-hibernation group and 60-d hibernation group (60S ribosomal protein L23, 60S ribosomal protein L26, 40S ribosomal protein S4, 60S ribosomal protein L11, 40S ribosomal protein S15a, 60S ribosomal protein L27a, 60S acidic ribosomal protein P2 isoform X3, 40S ribosomal protein S13, 60S ribosomal protein L10-like isoform X1, 40S ribosomal protein S25, 60S ribosomal protein L22, 40S ribosomal protein S7, Fig. 5).

**Fig. 6:**
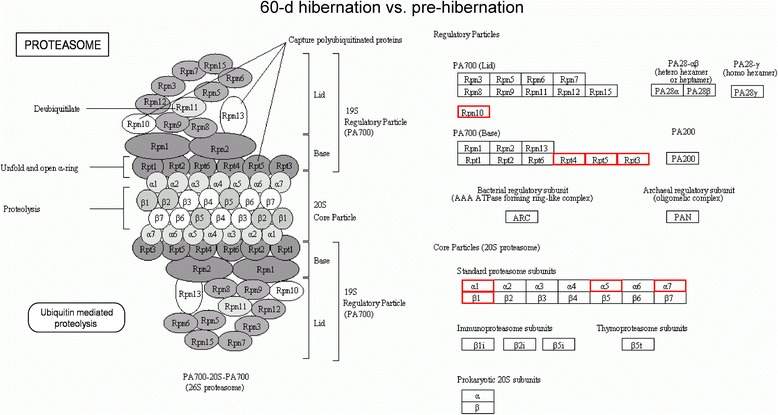
Pathway analysis using the KEGG Pathway database. Differentially expressed proteins enriched in the proteasome pathway in the 60-d hibernation ground squirrels group compared to the pre-hibernation group. Red indicates up-regulated expressed proteins. Rpn10: gi|532091148, 26S proteasome non-ATPase regulatory subunit 4; Rpt4: gi|59800155, 26S protease regulatory subunit 10B; Rpt5: gi|532088665, 26S protease regulatory subunit 6A; Rpt3: gi|532101732, 26S protease regulatory subunit 6B isoform X1; α1: gi|532073955, proteasome subunit alpha type-6; α5: gi|532105099, proteasome subunit alpha type-5; α7: gi|532073989, proteasome subunit alpha type-3 isoform X1. β1: gi|532103645, proteasome subunit beta type-6 isoform X1

**Fig. 7:**
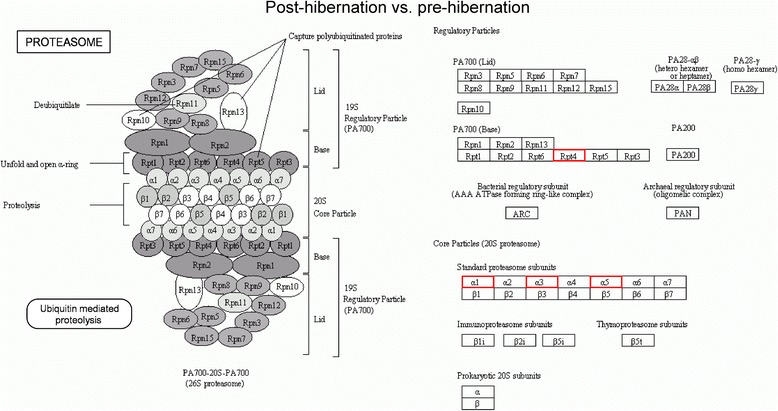
Pathway analysis using the KEGG Pathway database. Differentially expressed proteins enriched in the proteasome pathway in the post-hibernation ground squirrels group compared to the pre-hibernation group. Red indicates up-regulated expressed proteins. Rpt4: gi|59800155, 26S protease regulatory subunit 10B; α1: gi|532073955, proteasome subunit alpha type-6; α3: gi|532062055, proteasome subunit alpha type-4; α5: gi|532105099, proteasome subunit alpha type-5

**Fig. 8:**
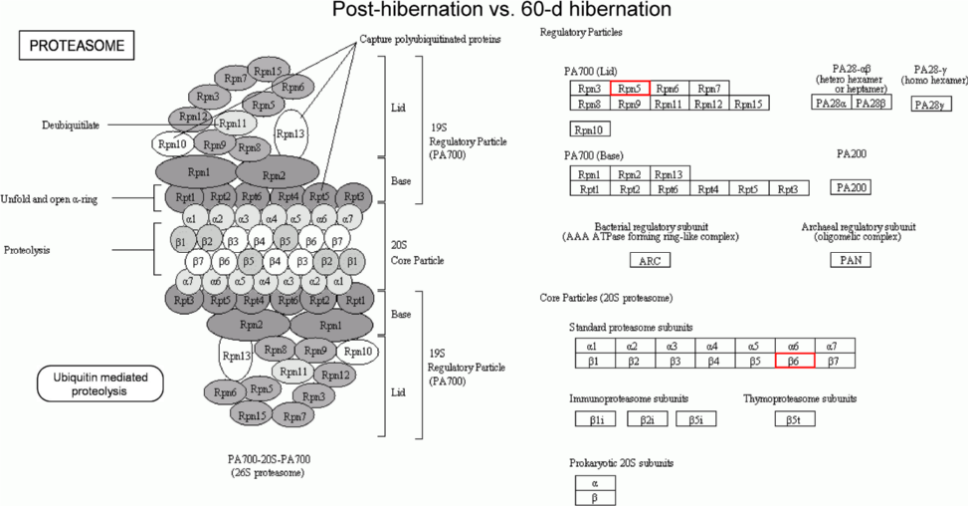
Pathway analysis using the KEGG Pathway database. Differentially expressed proteins enriched in the proteasome pathway in the post-hibernation ground squirrels group compared to the 60-d hibernation group. Red indicates up-regulated expressed proteins. Rpn5: gi|532085114, 26S proteasome non-ATPase regulatory subunit 12 isoform X1; β6: gi|532116708, proteasome subunit beta type-1

Numerous proteins were also involved in protein degradation. Proteasome is a multicatalytic proteinase complex with a highly ordered ring-shaped 20S core structure and distributed throughout eukaryotic cells at a high concentration and cleave peptides in an ATP-dependent process [26]. We found that 8 up-regulated proteins enriched in the proteasome pathway between 60-d hibernation group and pre-hibernation group (26S proteasome non-ATPase regulatory subunit 4, 26S protease regulatory subunit 10B, 26S protease regulatory subunit 6A, 26S protease regulatory subunit 6B isoform X1, proteasome subunit alpha type-6, proteasome subunit alpha type-5, proteasome subunit alpha type-3 isoform X1, proteasome subunit beta type-6 isoform X1, Fig. 6), 4 up-regulated proteins enriched in the proteasome pathway between post-hibernation group and pre-hibernation group (26S protease regulatory subunit 10B, proteasome subunit alpha type-6, proteasome subunit alpha type-4, proteasome subunit alpha type-5, Fig. 7), and 2 up-regulated proteins enriched in this category between post-hibernation group and 60-d -hibernation group (26S proteasome non-ATPase regulatory subunit 12 isoform X1, proteasome subunit beta type-1, Fig. 8).

### Validation of the quantitative proteomic analysis by Western blotting

**Fig. 9:**
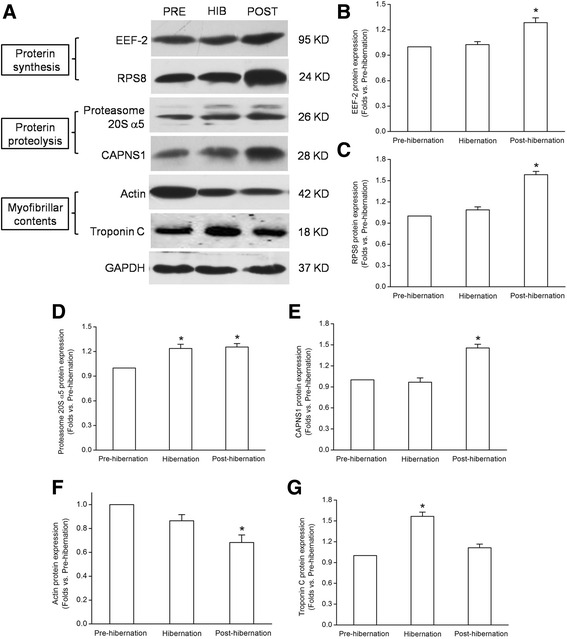
Western blot analysis of 6 differentially expressed proteins. **a** Representative bands of EEF-2, RPS8, proteasome 20S α5, CAPNS1, actin and troponin C in the soleus of pre-hibernation (PRE), hibernation (HIB) and post-hibernation (POST) squirrels. **b**-**g** Summarized data of protein quantity normalized by GAPDH in different groups. Values are mean ± SEM. *P < 0.05 versus pre-hibernation group

Six proteins (EEF-2 and RPS8 associated with proterin synthesis, proteasome 20S α5 and CANPS1 associated with proterin proteolysis, actin and troponin C associated with myofibrillar contents) with the marked differences in expression determined by iTRAQ based quantitative analysis were selected to be verified by western blot analysis. As shown in Fig. 9, EEF-2, RPS8, proteasome 20S α5 and CNPNS1 were up-regulated (*P* < 0.05) in post-hibernation group as compared with pre-hibernation group, and proteasome 20S α5 and troponin C were up-regulated (*P* < 0.05) in hibernation group as compared with pre-hibernation group. However, actin was down-regulated in post-hibernation group as compared with pre-hibernation group (*P* < 0.05), which is consistent with the findings in iTRAQ analysis.

### Determination of protein proteolytic rate

**Fig. 10:**
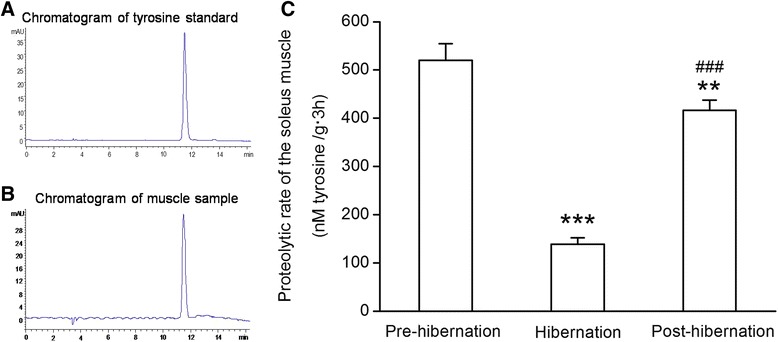
**a**: Representative chromatogram of tyrosine standard. **b**: Representative chromatogram of muscle sample. **c**: Total protein proteolytic rate of the soleus muscle in ground squirrels from pre-hibernation, 60-d and post-hibernation groups. Values are means ± SEM, *n* = 6 in each group. **P < 0.01 and *** *P* < 0.001 versus pre-hibernation group. ###*P* < 0.001 versus hibernation group

To determine whether the anti-muscle atrophy in the soleus of hibernating ground squirrels was a result of decreased proteolysis, we measured protein degradation by the release of the essential amino acid tyrosine. The rates of protein degradation decreased by 73 % (*P* < 0.001) in hibernation group as compared with the pre-hibernation group. Although the proteolytic rate in post-hibernation squirrels increased approximately 200 % (*P* < 0.001) as compared with the hibernation group, it was still 20 % (*P* < 0.01) lower than the pre-hibernation group (Fig. 10).

## Discussion

In present study, we collected the SOL muscle from 60-d and post-hibernation group with 2 months or more than 3 months of hibernation inactivity, which was long enough to cause significant atrophy in SOL muscle of non-hibernators [15]. The SOL muscle wet weight decreased less than the body weight in hibernating ground squirrels, which suggested an anti-atrophy effect during hibernation. Proteomic analysis was performed to investigated the protective proteins changes in soleus muscle of ground squirrels.

### Protective remodeling of myofibrillar proteins in preventing atrophy in SOL of Daurian ground squirrels during hibernation inactivity

The cytoplasm of a myofiber contains a regular array of contractile units (sarcomeres) comprised of actin-containing thin filaments and myosin-containing thick filaments that, along with additional regulatory and structural proteins, are arranged longitudinally as myofibrils [27]. With iTRAQ approach, we identified myosin-2 significantly up-regulated while myosin-3 significantly down-regulated in the 60-d hibernation group compared to the pre-hibernation group, meanwhile, myosin-13 significantly down-regulated in the post-hibernation group compared to the pre-hibernation group. Moreover, actin exhibited significantly down-regulated expression in the post-hibernation group compared to the pre-hibernation group (Table 3). The ratio of actin to myosin is one of the muscular atrophy hallmarks [28]. After 60-d hibernation inactivity, the ratio of actin/myosin filaments was likely to remain steady, however, a significant loss of myofibrillar proteins occured in post-hibernation group. Inconsistent with our results, the level of one isoform of actin was significantly higher (*P* < 0.05) in hibernation group than in summer active group in pectoral or biceps brachii muscles of the bat *Murina leucogaster* [28], which might be due to that the pectoral or biceps brachii muscles are involved in flight. But compared with the non-hibernator, lost of myofibrillar proteins in Daurian ground squirrel in hibernation is limited. For example, after 3 weeks of hindlimb unloading, levels of contractile proteins decreased by 40–70 %, and the ratio of actin/myosin filaments decreased by 31 % [8]. Reduction in muscle quality caused by alterations in myofilament contractile proteins (myosin and actin) may scale up from the molecular to the single fiber and tissue level to impact muscle performance [29]. Thus, up-regulation of myosin-2 is one of the most important mechanisms to maintain the integrity of the SOL muscle fiber in the 60-d hibernation ground squirrels. Because the function of myosin-3 and myosin-13 in hibernation is unknown, the relationship between the decrease of these two myosin subtypes and skeletal muscle function is not clarified.

Other regulatory proteins also showed significant changes in hibernation. Troponin C was found significantly up-regulated while tropomodulin-1 down-regulated in the 60-d hibernation group compared to the pre-hibernation group. However, both troponin C and tropomodulin-1 was unaltered in the post-hibernation group. The binding of Ca^2+^ to troponin C induces a series of conformational changes in troponin complex and sarcomeric actin thin filament to activate cross bridge cycling between myosin and actin and muscle contraction [30]. Troponin C increased by 59 % in soleus of human bed rest study [31], which indicated that troponin C in SOL of hibernation changed similarly as in disuse. Another regulatory protein tropomodulin, which is the only protein known to cap the pointed end of actin filaments, plays an important role in actin-driven processes by controlling the addition and dissociation of actin subunits at filament ends [32]. Calpain-mediated proteolysis of tropomodulin isoforms leads to thin filament elongation in dystrophic skeletal muscle [33]. It appears reasonable to assume that the changes of tropomodulin might be an adaptive factor for inhibiting the contractile activity during hibernation. Moreover, over-expression of tropomodulin-1 in mouse hearts results in degenerating myofibrils [34]. Therefore, we assume that the down-regulated tropomodulin-1 in 60-d hibernation might be a crucial component for regulating the length of actin-containing thin filament in soleus during hibernation.

Sarcomeric structural proteins such as α-actinin, titin, tropomyosin and desmin were not detected proteomic variations in present study, which supported our previous findings that the stable expression of atrogin-1 and MuRF1 may facilitate to prevent SOL atrophy via controlling ubiquitination of muscle proteins during hibernation [20, 21]. However, evidence showed that long-term disuse causes preferential loss of the giant sarcomere protein titin results in altered muscle function via abnormal sarcomeric organization [35]. The level of the T2 fragments of titin was observed decreased in the skeletal muscles of hibernating brown bear (*Ursidae, Mammalia*) [36]. Absence of α-actinin-3 resulted in reduced atrophic response and altered adaptation to disuse [37]. Desmin and titin globally reduced in hibernating myocardium suggested a qualitative cardiomyocyte remodeling [38]. Obviously, homeostasis of most sarcomeric structural proteins is an important mechanism against disuse atrophy in ground squirrels.

In conclusion, these results suggested that myofibrillar protective remodeling marked by dysregulated contractile proteins (myosins and actin) and regulatory proteins (troponin and tropomodulin), and maintain of most sarcomeric structural proteins is a major factor in protecting atrophy in SOL of Daurian ground squirrels during hibernation.

### The role of protein synthesis and proteolysis in preventing atrophy in SOL of Daurian ground squirrels during hibernation

Protein balance in skeletal muscles is a delicate interplay between protein synthesis and degradation [39]. High degradation and low synthesis of the proteins is known to cause significant loss of myofibrillar contents in most non-hibernators, including humans, prolonged disuse of skeletal muscle, as seen in bed rest, hindlimb suspension or spaceflight [40]. This study found that the number of up-regulated proteins was much higher in post-hibernation (248) than that of the proteins in 60-d hibernation group (96). Moreover, most of up-regulated proteins were mainly involved in protein binding, catalytic activity, transporter activity, enzyme regulator activity and other metabolic processes, which might be due to that the ground squirrels are recovering and likely produce these types of proteins rapidly upon resuming feeding and activity in 2 days awake after post-hibernation. These results indicated that protein synthesis was even strengthened in late hibernation period, which related to the regulation of contractile function. Our results agree well with the previous findings that the increase of protein synthesis to preserve and augment muscle mass in late winter was observed through direct measurements of protein synthesis by the in vivo SUnSET technique [41], which concurs with the finding here of accelerated synthesis in the later hibernation time point.

Here, we showed that there were 3 and 10 proteins with protein synthesis function significantly up-regulated in the 60-d hibernation group and post-hibernation group, respectively, relative to the pre-hibernation group (Fig. 2). Specifically, 60S ribosomal protein L39-like which was a structural constituent of ribosome participated in RNA binding and translation bioprocess, and polyadenylate-binding protein 4 isoform X1, which was a RNA-binding protein and locates in cytoplasmic stress granule and nucleus participated in RNA catabolic process and translation, were both significantly increased in the 60-d hibernation group compared to the pre-hibernation group. Besides, all of the up-regulated proteins were involved in translation, 7 of which were structural constituent of ribosome. In addition, elongation factor 1-beta isoform *X*2 which participates in translational elongation biological process was found continuously up-regulated in 60-d and post-hibernation groups relative to the pre-hibernation ground squirrels (Table 1).

In fact, down-regulated 40S ribosomal protein subunits in the 60-d hibernation ground squirrels, which bind to mRNA and modulate of the initiation phase of mRNA translation [42], suggested that the initiation of translation in protein synthesis was inhibited to a certain extent in hibernation. Consistent with our study, previous report demonstrated that mice homozygous for translation elongation factor 1A (eEF1A) deletion in muscle corresponds precisely to the onset of the wasted phenotype, characterized by muscle atrophy [43]. In addition, another serine-threonine kinase, serum- and glucocorticoid-regulated kinase 1 (SGK1), was upregulated during hibernation in 13-lined ground squirrel (*Ictidomys tridecemlineatus*) and contributed to protection from loss of muscle mass via an increased protein synthesis [44]. Hibernation factors including RMF (ribosome modulation factor), HPF (hibernation promoting factor) and YfiA (protein which inactivates ribosomes as 70S monomers) turn off protein synthesis via binding to the ribosome [45]. However, since the protein synthesis is a process of energy dissipation and the hibernating animals live in low temperature, fasting, or eating less state, we consider that the theory that the hibernating animals during prolonged period of immobilization and starvation by promoting protein synthesis to overcome muscular atrophy may not occupy a dominant position. Indeed, the rate of protein synthesis in vivo in the brain of torpid ground squirrels was just 0.04 % of that in active squirrels [46]. Together these findings suggest that protein synthesis was not inhibited during prolonged hibernation, which might play an important role in preventing atrophy in SOL of ground squirrels during hibernation.

The rates of protein degradation decreased significantly in hibernation and post-hibernation group as compared with the pre-hibernation group (Fig. 10). Different protein degradation pathways may be involved in sarcomeric protein loss in muscle atrophy. The ubiquitin-proteasome system and the autophagy-lysosome pathway are the major protein degradation systems involved in this process [47]. Moreover, calpains, a family of Ca^2+^-dependent proteases, play an initiating role in the protein degradation process and cause rapid and complete loss of Z-disk while activated by calcium.

In this study, we investigated that 10 and 14 proteins with protein proteolysis function were found significantly up-regulated in the 60-d hibernation group and post-hibernation group, respectively, relative to the pre-hibernation group (Fig. 2). Two-thirds of the dysregulated proteins were involved in ubiquitin-proteasome pathway and calpains pathway. Besides, proteasome subunit alpha type-5, proteasome subunit alpha type-6 and 26S protease regulatory subunit 10B were found continuously up-regulated in the 60-d and post-hibernation groups relative to the pre-hibernation ground squirrels (Table 2). These results indicated that ubiquitin-proteasome proteolysis was enhanced during prolonged hibernation. Actin, myosin heavy chains (in atrophying skeletal muscle), myosin light chains, members of the troponin family and telethonin were confirmed to be the ubiquitin-proteasome system substrates [48]. Telethonin, a substrate of the ubiquitin-proteasome system, was significantly down-regulated in the 60-d hibernation group compared to the pre-hibernation group. It is noteworthy that ubiquitin carboxyl-terminal hydrolase isozyme L3 was 1.591-fold increase in post-hibernation ground squirrels compared to pre-hibernation group. Similar with non-hibernators, the ubiquitin carboxy-terminal hydrolase L1 (UCHL1), functioning as an ubiquitin ligase and a mono-ubiquitin stabilizer, were also up-regulated in mice during hindlimb unloading [14], which suggest that ubiquitin carboxyl-terminal hydrolase might be activated in disuse. However, torpor may limit proteolysis in accordance with lower metabolic demands in livers of hibernating golden-mantled ground squirrels (*Spermophilus lateralis*) [49]. Importantly, calpastatin, an endogenous inhibitor of calpains, was found significantly up-regulated in the post-hibernation ground squirrels compared to the pre-hibernation group in present study, which is in consistent with our previous experimental report, in which the protein expression validation of calpastatin had been detected by western blot analyses, demonstrating the same result that inhibition of calpain activity and consequently calpain-mediated protein degradation by highly elevated calpastatin protein expression levels may be an important mechanism for preventing muscle protein loss during hibernation [15]. Nevertheless, calpain small subunit 1 also showed a significant increase in the post-hibernation ground squirrels compared to the pre-hibernation group from proteomic analysis. Calpains are heterodimers containing an identical 28-kDa regulatory small subunit and a distinct 80-kDa catalytic large subunit. Effective inhibition of the calpains by calpastatin requires that calpastatin binds specifically to the domain II or IV of large subunit of calpains in a Ca^2+^ dependent manner [50]. Hence, it appears reasonable to assume that calpains are inhibited at the level of enzymatic activity rather than the protein expression, and the increase of calpains expression make it possible that hibernating ground squirrels can get energy via the proteolysis of myofibrillar proteins to get through the energy crisis, while the degree of protein proteolysis can be also regulated by the enzymatic activity when squirrels do not need the muscle protein supply energy, thereby minimizing the muscular atrophy of disuse.

Collectively, these results suggested that the ubiquitin–proteasome catabolic pathway might be strengthened and be responsible for limited atrophy in the late hibernating period of Daurian ground squirrels. Moreover, calpains pathway might be regulated via the enzyme activity in hibernating ground squirrels, which subsequently regulate the ubiquitin–proteasome catabolic pathway and even the level of protein proteolysis of the whole body. Although recent study emphasizes autophagy-lysosome as another important proteolytic pathway in triggering the early stages of atrophy [47], no protein related to autophagy-lysosome pathway was detected proteomics differentially expressed in our study. Taken together, our study suggested that the limited SOL atrophy happened in the late hibernating period might be the result of activation of ubiquitin–proteasome catabolic pathway, and the effect of protecting atrophy in SOL during hibernation might be due to the inhibition of calpains and autophagy-lysosome proteolytic pathways.

## Conclusions

In this study, we present a proteomic analysis in soleus muscle of Daurian ground squirrels for the first time. These findings not only provide novel insights into the myofibrillar remodeling, which was marked by dysregulated contractile proteins (myosins and actin) and regulatory proteins (troponin and tropomodulin) and maintain of most sarcomeric structural proteins, contributes to a protective effect that prevents muscle atrophy in spite of prolonged disuse during hibernation, but also provide the first experimental evidence that the total proteolysis rates of soleus in hibernating ground squirrels is decreased. Moreover, the strengthened ubiquitin-proteasome pathway and calpains pathway related to the protein degradation, associated with higher level of calpastatin, contributes to maintain of most myofibrillar proteins in hibernation. Although the number of differentially expressed proteins associated with protein degradation is much more than those associated with protein synthesis in hibernation, the increased part may be partially offset by the strengthened ubiquitin–proteasome catabolic pathway,which together with partial inhibition of calpains might play a critical role in maintain of myofibrillar proteins and provide a foundation for elucidating the mechanisms of prevention of the disuse atrophy in skeletal muscle in non-hibernation animals.

### Limitations of the study

The iTRAQ-based proteomic analysis should have biological triplicates, however, we have validated the expression levels of some proteins using western blot technology to make up for this deficiency. In addition, iTRAQ-based quantitative proteomic analysis does not involve the differentially modulation of post-translational modification of proteins which are critical in adaptation to hibernating state. Besides, the proteins and pathways identified by computer-based statistical algorithms also need to be verified experimentally in future.

## Additional file

Additional file 1: Table S1. Summary table showing a full list of identified proteins and their relative expression in SOL muscle among pre-hibernation, 60-d hibernation and 112-d hibernation groups in Daurian ground squirrels identified by iTRAQ. (DOCX 101 kb)
