# Supplementary material for: iTRAQ-based proteomic analysis of myofibrillar contents and relevant synthesis and proteolytic proteins in soleus muscle of hibernating Daurian ground squirrels (Spermophilus dauricus)
Source: Proteome Sci. 2016 Nov 8;14:16. doi: 10.1186/s12953-016-0105-x (PMC5101720; doi:10.1186/s12953-016-0105-x)
Supplement: Additional file 1: Table S1. — Summary table showing a full list of identified proteins and their relative expression in SOL muscle among pre-hibernation, 60-d hibernation and 112-d hibernation groups in Daurian ground squirrels identified by iTRAQ. (DOCX 101 kb) [file 12953_2016_105_MOESM1_ESM.docx]

**Supplementary material**

**Table.5** Summary table showing a full list of identified proteins and their relative expression in SOL muscle among pre-hibernation, 60-d hibernation and 112-d hibernation groups in Daurian ground squirrels identified by iTRAQ.

Prot name (protein name), MW (Molecular mass), Sig (**P*-value < 0.05 was considered statistically significant).

| 60-d hibernation  vs  pre-hibernation | | | 112-d hibernation  vs  pre-hibernation | | | 112-d hibernation  vs  60-d hibernation | | |
| --- | --- | --- | --- | --- | --- | --- | --- | --- |
| Protein name | Fold | Sig | Protein name | Fold | Sig | Protein name | Fold | Sig |
| **Up-regulated** | | | | | | | | |
| elongation factor 1-beta isoform X2 | 1.348 | * | calpastatin | 1.226 | * | profilin-1 | 1.341 | * |
| mitochondrial-processing peptidase subunit alpha | 1.218 | * | transthyretin | 2.743 | * | transthyretin | 2.62 | * |
| fibromodulin | 1.865 | * | myosin-binding protein H | 1.696 | * | 60S ribosomal protein L11 | 1.329 | * |
| heat shock protein beta-2 isoform X2 | 1.239 | * | ELAV-like protein 1 isoform X1 | 1.243 | * | activated RNA polymerase II transcriptional coactivator p15 | 1.367 | * |
| 60S ribosomal protein L39-like | 1.791 | * | ATP-dependent RNA helicase DDX3X | 1.283 | * | alpha/beta hydrolase domain-containing protein 11 isoform X1 | 1.593 | * |
| 26S proteasome non-ATPase regulatory subunit 5 isoform X1 | 1.517 | * | protein disulfide-isomerase A3 | 1.391 | * | protein S100-A1 isoform X2 | 1.339 | * |
| myelin basic protein | 1.456 | * | thioredoxin isoform X1 | 1.37 | * | band 4.1-like protein 2 | 1.637 | * |
| thioredoxin isoform X1 | 1.231 | * | activated RNA polymerase II transcriptional coactivator p15 | 1.467 | * | transgelin-2 | 1.887 | * |
| 26S proteasome non-ATPase regulatory subunit 4 | 1.488 | * | carbonic anhydrase 2 | 1.648 | * | transketolase | 1.621 | * |
| protein-glutamine gamma-glutamyltransferase 2 | 1.547 | * | myc box-dependent-interacting protein 1 isoform X1 | 1.225 | * | hepatoma-derived growth factor isoform X1 | 1.265 | * |
| proteasome subunit beta type-6 isoform X1 | 1.295 | * | spectrin alpha chain, erythrocytic 1 isoform X1 | 3.427 | * | ras-related C3 botulinum toxin substrate 1 | 1.296 | * |
| carbonic anhydrase 2 | 1.258 | * | coiled-coil-helix-coiled-coil-helix domain-containing protein 10, mitochondrial | 1.625 | * | 40S ribosomal protein S25 | 1.228 | * |
| calsequestrin-2 | 1.275 | * | hydroxyacyl-coenzyme A dehydrogenase, mitochondrial isoform X1 | 1.279 | * | fibrinogen gamma chain | 3.349 | * |
| cold-inducible RNA-binding protein isoform X1 | 1.354 | * | endoplasmic reticulum resident protein 44 | 1.659 | * | aspartate aminotransferase, mitochondrial | 1.344 | * |
| tRNA-splicing ligase RtcB homolog | 1.287 | * | fibrinogen gamma chain | 2.229 | * | calreticulin | 1.362 | * |
| retinol-binding protein 4 | 1.57 | * | calreticulin | 1.831 | * | electron transfer flavoprotein subunit beta, partial | 1.344 | * |
| calreticulin | 1.42 | * | calmodulin | 1.686 | * | ribose-phosphate pyrophosphokinase 1 | 1.413 | * |
| hypoxanthine-guanine phosphoribosyltransferase | 1.286 | * | collagen alpha-2(VI) chain isoform X1 | 1.263 | * | calmodulin | 1.441 | * |
| proteasome subunit alpha type-3 isoform X1 | 1.209 | * | alanine aminotransferase 2 | 1.662 | * | aspartate aminotransferase, cytoplasmic | 1.234 | * |
| microfibrillar-associated protein 5 | 1.492 | * | collagen alpha-2(I) chain isoform X1 | 3.401 | * | macrophage-capping protein | 1.543 | * |
| protein phosphatase 1 regulatory subunit 12B isoform X1 | 1.236 | * | creatine kinase B-type | 1.672 | * | collagen alpha-2(I) chain isoform X1 | 5.38 | * |
| PRA1 family protein 3 | 1.227 | * | putative RNA-binding protein 3-like | 2.38 | * | creatine kinase B-type | 1.367 | * |
| serine/threonine-protein kinase OSR1 isoform X1 | 1.429 | * | glutathione S-transferase alpha-3-like | 1.239 | * | putative RNA-binding protein 3-like | 1.796 | * |
| adenylate kinase 2, mitochondrial isoform X1 | 1.309 | * | zyxin | 1.371 | * | small muscular protein isoform X2 | 1.28 | * |
| small muscular protein isoform X2 | 1.238 | * | small muscular protein isoform X2 | 1.392 | * | glutathione peroxidase 1 | 1.289 | * |
| sorbin and SH3 domain-containing protein 1 | 1.219 | * | fructose-bisphosphate aldolase C | 1.256 | * | coagulation factor XIII A chain | 1.42 | * |
| delta-aminolevulinic acid dehydratase isoform X1 | 1.255 | * | tubulin alpha-8 chain isoform X1 | 1.355 | * | transgelin isoform X2 | 1.389 | * |
| dnaJ homolog subfamily A member 4 isoform X1 | 1.527 | * | endoplasmin | 1.316 | * | aldehyde dehydrogenase, mitochondrial isoform X1 | 1.608 | * |
| phospholipase A-2-activating protein | 1.214 | * | cytochrome c oxidase subunit II | 1.697 | * | 5~-AMP-activated protein kinase catalytic subunit alpha-2 | 1.478 | * |
| apolipoprotein C-II isoform X2 | 2.078 | * | 40S ribosomal protein S8 | 1.349 | * | thioredoxin reductase 2, mitochondrial | 1.394 | * |
| 2-oxoisovalerate dehydrogenase subunit alpha, mitochondrial | 1.234 | * | serpin H1 isoform X4 | 1.981 | * | 60S ribosomal protein L22 | 1.364 | * |
| NSFL1 cofactor p47 isoform X1 | 1.221 | * | lipoma-preferred partner isoform X1 | 1.36 | * | serpin H1 isoform X4 | 2.751 | * |
| dnaJ homolog subfamily A member 2 | 1.232 | * | guanidinoacetate N-methyltransferase | 1.416 | * | eukaryotic initiation factor 4A-II isoform X1 | 1.236 | * |
| cofilin-1 | 1.239 | * | eukaryotic initiation factor 4A-II isoform X1 | 1.232 | * | cofilin-1 | 1.27 | * |
| nidogen-2 isoform X1 | 1.22 | * | cadherin-13 | 1.715 | * | nidogen-2 isoform X1 | 1.34 | * |
| cytosol aminopeptidase | 1.202 | * | cofilin-1 | 1.535 | * | COP9 signalosome complex subunit 8 | 1.502 | * |
| polyadenylate-binding protein 4 isoform X1 | 1.508 | * | F-actin-capping protein subunit beta isoform X2 | 1.219 | * | catalase | 1.455 | * |
| glutathione S-transferase Mu 2-like isoform X4 | 1.347 | * | ubiquinone biosynthesis protein COQ7 homolog | 1.336 | * | heterogeneous nuclear ribonucleoproteins A2/B1 isoform X1 | 1.328 | * |
| catalase | 1.21 | * | dnaJ homolog subfamily B member 11 | 1.346 | * | ras-related protein Rab-11A | 1.214 | * |
| keratin, type II cytoskeletal 7 | 2.166 | * | nidogen-2 isoform X1 | 1.467 | * | alcohol dehydrogenase class-3 | 1.312 | * |
| nebulin-related-anchoring protein | 1.211 | * | histone H1.0-like | 1.784 | * | hsc70-interacting protein isoform X1 | 1.247 | * |
| proteasome subunit alpha type-6 | 1.23 | * | COP9 signalosome complex subunit 8 | 1.59 | * | biglycan | 2.364 | * |
| chromobox protein homolog 3 | 1.344 | * | catalase | 1.766 | * | myozenin-3 | 1.233 | * |
| Hemoglobin subunit beta-S/F | 1.627 | * | heterogeneous nuclear ribonucleoproteins A2/B1 isoform X1 | 1.392 | * | asporin isoform X2 | 1.628 | * |
| apolipoprotein A-I | 1.313 | * | ras-related protein Rab-11A | 1.384 | * | actin-related protein 3 isoform X1 | 1.337 | * |
| V-type proton ATPase subunit B, brain isoform | 1.283 | * | alcohol dehydrogenase class-3 | 1.344 | * | 60S ribosomal protein L23 | 1.345 | * |
| ruvB-like 2 | 1.465 | * | ras-related protein Rab-5A | 1.516 | * | guanine nucleotide-binding protein subunit beta-2-like 1 | 1.242 | * |
| protein CutA isoform X2 | 1.297 | * | chromobox protein homolog 3 | 1.585 | * | myosin light polypeptide 6 isoform X1 | 1.338 | * |
| 26S protease regulatory subunit 6B isoform X1 | 1.246 | * | ubiquitin-like modifier-activating enzyme 1 isoform X2 | 1.242 | * | protein unc-45 homolog B | 1.361 | * |
| proteasome subunit alpha type-5 | 1.226 | * | calcyclin-binding protein | 1.457 | * | tetratricopeptide repeat protein 38 | 1.777 | * |
| cAMP-dependent protein kinase type I-alpha regulatory subunit | 1.292 | * | biglycan | 1.91 | * | histone H2B type 1-M-like | 1.551 | * |
| myosin-2 | 2.135 | * | myozenin-3 | 1.401 | * | myosin regulatory light chain 12B | 1.324 | * |
| inosine-5~-monophosphate dehydrogenase 2 isoform X1 | 1.229 | * | asporin isoform X2 | 1.795 | * | hydroxyacylglutathione hydrolase, mitochondrial isoform X1 | 1.212 | * |
| heterogeneous nuclear ribonucleoprotein L isoform X1 | 1.277 | * | proliferation-associated protein 2G4 | 1.206 | * | COP9 signalosome complex subunit 4 isoform X2 | 1.391 | * |
| vimentin | 1.211 | * | guanine nucleotide-binding protein subunit beta-2-like 1 | 1.231 | * | calnexin | 1.298 | * |
| early endosome antigen 1 | 1.288 | * | myosin light polypeptide 6 isoform X1 | 1.576 | * | hemopexin | 1.784 | * |
| leiomodin-3 isoform X2 | 1.371 | * | V-type proton ATPase subunit B, brain isoform | 1.401 | * | Hemoglobin subunit alpha | 1.749 | * |
| transcription factor A, mitochondrial | 1.381 | * | tetratricopeptide repeat protein 38 | 1.942 | * | glutamine--tRNA ligase isoform X1 | 1.338 | * |
| kynurenine--oxoglutarate transaminase 1 | 1.429 | * | moesin-like | 1.248 | * | xaa-Pro aminopeptidase 1-like isoform X1 | 1.43 | * |
| PDZ and LIM domain protein 3 isoform X1 | 1.304 | * | histone H2B type 1-M-like | 1.26 | * | histone H3.3 | 1.351 | * |
| methylcrotonoyl-CoA carboxylase beta chain, mitochondrial | 1.417 | * | 78 kDa glucose-regulated protein isoform X2 | 1.429 | * | decorin isoform X2 | 1.428 | * |
| ATP synthase subunit delta, mitochondrial isoform X1 | 1.232 | * | protein CutA isoform X2 | 1.411 | * | adenylyl cyclase-associated protein 1 isoform X2 | 1.34 | * |
| protein FAM98B | 1.411 | * | myosin regulatory light chain 12B | 1.771 | * | talin-1 | 1.302 | * |
| 2-oxoisovalerate dehydrogenase subunit beta, mitochondrial | 2.589 | * | hydroxyacylglutathione hydrolase, mitochondrial isoform X1 | 1.238 | * | heterogeneous nuclear ribonucleoprotein H2 isoform X3 | 1.411 | * |
| pyruvate kinase PKM isoform X1 | 1.253 | * | importin subunit alpha-4 | 1.209 | * | collagen alpha-1(XII) chain | 2.479 | * |
| leucine-rich repeat-containing protein 47 | 1.246 | * | glucose-6-phosphate isomerase isoform X1 | 1.237 | * | fibulin-5 | 1.795 | * |
| prolargin | 1.309 | * | COP9 signalosome complex subunit 4 isoform X2 | 1.626 | * | 60S ribosomal protein L27a | 1.214 | * |
| 26S protease regulatory subunit 10B | 1.281 | * | cAMP-dependent protein kinase type I-alpha regulatory subunit | 1.378 | * | cathepsin D | 1.567 | * |
| hematopoietic prostaglandin D synthase | 1.45 | * | periaxin | 1.753 | * | apolipoprotein D | 3.094 | * |
| heterogeneous nuclear ribonucleoprotein A1 isoform X1 | 1.495 | * | phosphoglycerate kinase 1-like | 1.285 | * | cat eye syndrome critical region protein 5, partial | 1.337 | * |
| alpha-1-acid glycoprotein-like | 1.535 | * | glutathione S-transferase kappa 1 isoform X1 | 1.315 | * | maleylacetoacetate isomerase isoform X1 | 1.371 | * |
| 26S protease regulatory subunit 6A | 1.32 | * | glutamine--tRNA ligase isoform X1 | 1.27 | * | immunoglobulin lambda-like polypeptide 5-like | 1.78 | * |
| mitochondrial import receptor subunit TOM34 | 1.506 | * | xaa-Pro aminopeptidase 1-like isoform X1 | 1.485 | * | Hemoglobin subunit alpha | 1.749 | * |
| bis(5~-nucleosyl)-tetraphosphatase | 1.266 | * | decorin isoform X2 | 1.827 | * | PDZ and LIM domain protein 7 | 1.591 | * |
| PDZ and LIM domain protein 1 | 1.467 | * | heterogeneous nuclear ribonucleoprotein H2 isoform X3 | 1.809 | * | prolargin | 2.353 | * |
| selenium-binding protein 1 | 1.375 | * | talin-1 | 1.288 | * | nucleoside diphosphate kinase B | 1.283 | * |
| protein S100-A6 | 1.404 | * | myosin regulatory light polypeptide 9 isoform X1 | 1.68 | * | eukaryotic translation initiation factor 1b | 1.218 | * |
| galectin-1 | 1.334 | * | filamin-A | 1.441 | * | fibrinogen alpha chain | 3.997 | * |
| methylmalonate-semialdehyde dehydrogenase | 1.272 | * | collagen alpha-1(XII) chain | 2.682 | * | protein disulfide-isomerase | 1.284 | * |
| D-dopachrome decarboxylase-like | 1.343 | * | fibulin-5 | 1.894 | * | Fatty acid-binding protein, adipocyte | 1.29 | * |
| translationally-controlled tumor protein | 1.281 | * | profilin-2-like isoform X1 | 1.246 | * | ras suppressor protein 1 | 1.265 | * |
| erythrocyte membrane protein band 4.2 | 2.597 | * | cathepsin D | 1.658 | * | 6-phosphogluconate dehydrogenase, decarboxylating | 1.265 | * |
| non-specific lipid-transfer protein-like isoform X1 | 1.351 | * | UPF0568 protein C14orf166 homolog | 1.714 | * | proteasome subunit beta type-1 | 1.283 | * |
| troponin C, slow skeletal and cardiac muscles | 1.358 | * | apolipoprotein D | 2.838 | * | protein S100-A10 | 1.512 | * |
| cAMP-dependent protein kinase type II-alpha regulatory subunit isoform X1 | 1.378 | * | alpha-protein kinase 3 isoform X1 | 1.272 | * | ankyrin-1 isoform X1 | 1.335 | * |
| tubulin polymerization-promoting protein family member 3 | 1.607 | * | maleylacetoacetate isomerase isoform X1 | 1.482 | * | glyoxalase domain-containing protein 4 | 1.292 | * |
| pyruvate carboxylase, mitochondrial isoform X2 | 1.256 | * | NADH dehydrogenase | 1.234 | * | uridine 5~-monophosphate synthase | 1.305 | * |
| exportin-2 isoform X1 | 1.367 | * | beta-taxilin | 1.384 | * | eukaryotic peptide chain release factor subunit 1 isoform X1 | 1.297 | * |
| serotransferrin isoform X1 | 1.402 | * | rab GDP dissociation inhibitor beta isoform X1 | 1.25 | * | protein S100-A6 | 1.563 | * |
| band 3 anion transport protein | 3.013 | * | COP9 signalosome complex subunit 6 isoform X1 | 1.298 | * | ferritin heavy chain | 2.049 | * |
| prothrombin | 1.944 | * | pyruvate kinase PKM isoform X1 | 1.495 | * | vitamin D-binding protein | 1.865 | * |
| myosin light chain 4 isoform X2 | 1.434 | * | alpha-2-macroglobulin-like | 1.449 | * | heterogeneous nuclear ribonucleoprotein U | 1.367 | * |
| LIM and cysteine-rich domains protein 1 isoform X1 | 1.214 | * | protein-L-isoaspartate(D-aspartate) O-methyltransferase isoform X1 | 1.376 | * | heterogeneous nuclear ribonucleoprotein A3 isoform X2 | 1.233 | * |
| acylphosphatase-2 | 1.397 | * | serine/threonine-protein phosphatase 2A 55 kDa regulatory subunit B alpha isoform isoform X1 | 1.246 | * | superoxide dismutase | 1.267 | * |
| heterogeneous nuclear ribonucleoprotein D-like isoform X2 | 1.34 | * | PDZ and LIM domain protein 7 | 1.653 | * | Actin, cytoplasmic 1 | 1.373 | * |
| isochorismatase domain-containing protein 2A, mitochondrial isoform X1 | 1.286 | * | 26S protease regulatory subunit 10B | 1.264 | * | 40S ribosomal protein S4, X isoform | 1.244 | * |
|  |  |  | prolargin | 3.085 | * | erythrocyte membrane protein band 4.2 | 1.434 | * |
|  |  |  | fructose-1,6-bisphosphatase isozyme 2 | 1.517 | * | transferrin | 1.595 | * |
|  |  |  | alpha-1-acid glycoprotein-like | 1.327 | * | UV excision repair protein RAD23 homolog A isoform X1 | 1.249 | * |
|  |  |  | hematopoietic prostaglandin D synthase | 1.465 | * | cysteine-rich protein 2 isoform X1 | 2.582 | * |
|  |  |  | 60S ribosomal protein L38 isoform X2 | 1.679 | * | lumican | 1.807 | * |
|  |  |  | Fatty acid-binding protein, adipocyte | 1.28 | * | antithrombin-III | 1.595 | * |
|  |  |  | ras suppressor protein 1 | 1.27 | * | ATP-dependent RNA helicase DDX1 | 1.305 | * |
|  |  |  | protein S100-A10 | 1.523 | * | tropomodulin-1 | 1.509 | * |
|  |  |  | ankyrin-1 isoform X1 | 2.69 | * | band 3 anion transport protein | 1.499 | * |
|  |  |  | serine/threonine-protein phosphatase 2A catalytic subunit alpha isoform | 1.293 | * | prothrombin | 1.58 | * |
|  |  |  | glyoxalase domain-containing protein 4 | 1.339 | * | serum paraoxonase/arylesterase 1 isoform X1 | 1.961 | * |
|  |  |  | uridine 5~-monophosphate synthase | 1.542 | * | coenzyme Q-binding protein COQ10 homolog A, mitochondrial isoform X1 | 1.305 | * |
|  |  |  | septin-2 | 1.361 | * | transforming growth factor-beta-induced protein ig-h3 | 1.523 | * |
|  |  |  | selenium-binding protein 1 | 1.256 | * | fatty acid-binding protein, epidermal-like | 1.261 | * |
|  |  |  | protein S100-A6 | 2.006 | * | heat shock protein beta-2 isoform X2 | 1.276 | * |
|  |  |  | heat shock-related 70 kDa protein 2 | 1.216 | * | fibromodulin | 2.778 | * |
|  |  |  | vitamin D-binding protein | 1.657 | * | complement C3-like, partial | 2.034 | * |
|  |  |  | heterogeneous nuclear ribonucleoprotein A3 isoform X2 | 1.497 | * | lactoylglutathione lyase | 1.321 | * |
|  |  |  | sorting nexin-3 isoform X1 | 1.321 | * | vitronectin | 3.095 | * |
|  |  |  | T-complex protein 1 subunit gamma isoform X1 | 1.223 | * | myelin basic protein | 2.662 | * |
|  |  |  | Actin, cytoplasmic 1 | 1.366 | * | ras-related protein Rab-7a | 1.205 | * |
|  |  |  | erythrocyte membrane protein band 4.2 | 3.653 | * | 26S proteasome non-ATPase regulatory subunit 12 isoform X1 | 1.457 | * |
|  |  |  | translationally-controlled tumor protein | 1.25 | * | bleomycin hydrolase | 1.427 | * |
|  |  |  | UV excision repair protein RAD23 homolog A isoform X1 | 1.434 | * | neurofilament, light polypeptide, partial | 1.528 | * |
|  |  |  | antithrombin-III | 1.922 | * | annexin A1 | 1.375 | * |
|  |  |  | ATP-dependent RNA helicase DDX1 | 1.26 | * | Pyruvate dehydrogenase | 1.48 | * |
|  |  |  | barrier-to-autointegration factor isoform X5 | 1.415 | * | thrombospondin-4 | 2.011 | * |
|  |  |  | myelin protein P0 | 1.949 | * | integrin alpha-7 | 1.556 | * |
|  |  |  | band 3 anion transport protein | 4.271 | * | aspartate aminotransferase, mitochondrial-like | 1.376 | * |
|  |  |  | 60S ribosomal protein L30-like | 1.366 | * | myocilin | 2.983 | * |
|  |  |  | prothrombin | 2.441 | * | adenylosuccinate synthetase isozyme 1 | 1.263 | * |
|  |  |  | medium-chain specific acyl-CoA dehydrogenase, mitochondrial | 1.227 | * | phosphoglycerate mutase 1 | 1.523 | * |
|  |  |  | myosin light chain 4 isoform X2 | 1.579 | * | actin-related protein 2/3 complex subunit 5 isoform X1 | 1.351 | * |
|  |  |  | acylphosphatase-2 | 1.835 | * | 40S ribosomal protein S13 | 1.238 | * |
|  |  |  | transforming growth factor-beta-induced protein ig-h3 | 1.379 | * | adiponectin isoform X2 | 1.255 | * |
|  |  |  | fatty acid-binding protein, epidermal-like | 1.494 | * | flavin reductase (NADPH), partial | 1.294 | * |
|  |  |  | elongation factor 1-beta isoform X2 | 1.202 | * | inter-alpha-trypsin inhibitor heavy chain H4 isoform X1 | 2.122 | * |
|  |  |  | heat shock protein beta-2 isoform X2 | 1.471 | * | histidine-rich glycoprotein | 1.679 | * |
|  |  |  | fibromodulin | 5.104 | * | Alpha-1-antitrypsin-like protein GS55-MS | 1.535 | * |
|  |  |  | complement C3-like, partial | 1.799 | * | transcriptional activator protein Pur-alpha | 1.45 | * |
|  |  |  | lactoylglutathione lyase | 1.475 | * | cystatin-B | 1.521 | * |
|  |  |  | vitronectin | 2.429 | * | phenylalanine--tRNA ligase beta subunit isoform X1 | 1.452 | * |
|  |  |  | transitional endoplasmic reticulum ATPase | 1.336 | * | 60S ribosomal protein L10-like isoform X1 | 1.419 | * |
|  |  |  | tubulin beta-5 chain-like | 1.367 | * | bisphosphoglycerate mutase | 1.764 | * |
|  |  |  | cytochrome c oxidase subunit 5A, mitochondrial-like | 1.238 | * | neurofilament heavy polypeptide | 1.72 | * |
|  |  |  | protein-glutamine gamma-glutamyltransferase 2 | 1.553 | * | importin subunit beta-1 isoform X1 | 1.314 | * |
|  |  |  | ubiquitin-60S ribosomal protein L40-like | 1.371 | * | neurofilament medium polypeptide | 1.669 | * |
|  |  |  | neurofilament, light polypeptide, partial | 1.456 | * | ankyrin repeat domain-containing protein 2 | 1.268 | * |
|  |  |  | Pyruvate dehydrogenase | 1.391 | * | 1,4-alpha-glucan-branching enzyme-like | 1.324 | * |
|  |  |  | thrombospondin-4 | 2.406 | * | tubulin beta-4B chain isoform X1 | 1.25 | * |
|  |  |  | hypoxanthine-guanine phosphoribosyltransferase | 1.372 | * | fibrillin-1 | 1.685 | * |
|  |  |  | aspartate aminotransferase, mitochondrial-like | 1.379 | * | peptidyl-prolyl cis-trans isomerase B | 1.494 | * |
|  |  |  | annexin A2 | 1.502 | * | prefoldin subunit 5 isoform X1 | 1.345 | * |
|  |  |  | myocilin | 2.837 | * | annexin A5 | 1.643 | * |
|  |  |  | mth938 domain-containing protein isoform X1 | 1.263 | * | dermatopontin | 1.578 | * |
|  |  |  | 40S ribosomal protein S13 | 1.363 | * | rho GDP-dissociation inhibitor 1 isoform X1 | 1.35 | * |
|  |  |  | flavin reductase (NADPH), partial | 1.539 | * | 40S ribosomal protein S7 | 1.212 | * |
|  |  |  | inter-alpha-trypsin inhibitor heavy chain H4 isoform X1 | 2.335 | * | 60S acidic ribosomal protein P2 isoform X3 | 1.265 | * |
|  |  |  | heat shock protein beta-7 isoform X1 | 1.217 | * | carbonic anhydrase 1 | 1.265 | * |
|  |  |  | glutathione S-transferase P | 1.305 | * | protein DJ-1 | 1.297 | * |
|  |  |  | cystatin-B | 1.598 | * | T-complex protein 1 subunit alpha | 1.246 | * |
|  |  |  | phenylalanine--tRNA ligase beta subunit isoform X1 | 1.412 | * | tubulin alpha-1D chain-like | 2.402 | * |
|  |  |  | 60S ribosomal protein L10-like isoform X1 | 1.276 | * | staphylococcal nuclease domain-containing protein 1 | 1.465 | * |
|  |  |  | bisphosphoglycerate mutase | 1.849 | * | smoothelin-like protein 2 | 1.922 | * |
|  |  |  | neurofilament heavy polypeptide | 1.55 | * | apolipoprotein A-I preproprotein | 2.077 | * |
|  |  |  | importin subunit beta-1 isoform X1 | 1.537 | * | protein S100-A4 | 2.302 | * |
|  |  |  | neurofilament medium polypeptide | 1.677 | * | neuroblast differentiation-associated protein AHNAK-like | 1.265 | * |
|  |  |  | 1,4-alpha-glucan-branching enzyme-like | 1.252 | * | laminin subunit gamma-1 | 1.281 | * |
|  |  |  | tubulin beta-4B chain isoform X1 | 1.366 | * | poly(rC)-binding protein 2 isoform X1 | 1.724 | * |
|  |  |  | tubulin alpha-4A chain | 1.396 | * | peptidyl-prolyl cis-trans isomerase FKBP3 | 1.386 | * |
|  |  |  | heterogeneous nuclear ribonucleoprotein K isoform X4 | 1.371 | * | heterogeneous nuclear ribonucleoprotein D0 | 1.25 | * |
|  |  |  | heterogeneous nuclear ribonucleoproteins C1/C2 | 1.432 | * | fibronectin isoform X1 | 1.742 | * |
|  |  |  | GTP:AMP phosphotransferase AK3, mitochondrial isoform X1 | 1.296 | * | vimentin | 1.828 | * |
|  |  |  | keratin, type II cytoskeletal 7 | 2.049 | * | platelet-activating factor acetylhydrolase IB subunit beta | 1.331 | * |
|  |  |  | peptidyl-prolyl cis-trans isomerase B | 1.324 | * | 6-phosphogluconolactonase | 1.549 | * |
|  |  |  | prefoldin subunit 5 isoform X1 | 2.048 | * | voltage-dependent L-type calcium channel subunit beta-1 isoform X3 | 1.327 | * |
|  |  |  | proteasome subunit alpha type-6 | 1.248 | * | heterogeneous nuclear ribonucleoprotein M isoform X1 | 1.227 | * |
|  |  |  | elongation factor 2 | 1.227 | * | chaperone activity of bc1 complex-like, mitochondrial isoform X3 | 1.328 | * |
|  |  |  | apolipoprotein A-I | 3.087 | * | apolipoprotein A-IV | 1.733 | * |
|  |  |  | annexin A5 | 1.302 | * | NADP-dependent malic enzyme, mitochondrial | 1.475 | * |
|  |  |  | rho GDP-dissociation inhibitor 1 isoform X1 | 1.414 | * | poly(rC)-binding protein 1 isoform X1 | 1.254 | * |
|  |  |  | pyruvate kinase PKM isoform X2 | 1.255 | * | isoleucine--tRNA ligase, mitochondrial | 1.264 | * |
|  |  |  | dermatopontin | 1.699 | * | calpain small subunit 1 | 1.488 | * |
|  |  |  | 60S acidic ribosomal protein P2 isoform X3 | 1.455 | * | plasminogen-like isoform X1 | 1.745 | * |
|  |  |  | T-complex protein 1 subunit alpha | 1.323 | * | transmembrane emp24 domain-containing protein 10 | 1.368 | * |
|  |  |  | tubulin alpha-1D chain-like | 2.103 | * | carboxymethylenebutenolidase homolog isoform X2 | 1.285 | * |
|  |  |  | staphylococcal nuclease domain-containing protein 1 | 1.324 | * | cartilage intermediate layer protein 2 | 4.521 | * |
|  |  |  | protein S100-A4 | 1.819 | * | hemoglobin subunit zeta | 3.508 | * |
|  |  |  | proteasome subunit alpha type-5 | 1.226 | * | unconventional myosin-Ic | 1.328 | * |
|  |  |  | protein 4.1 isoform X1 | 2.552 | * | elongation factor 1-delta isoform X4 | 1.292 | * |
|  |  |  | laminin subunit gamma-1 | 1.271 | * | cytochrome c, somatic-like | 1.408 | * |
|  |  |  | mitochondrial import inner membrane translocase subunit Tim13 | 1.231 | * | eukaryotic translation initiation factor 3 subunit F | 1.262 | * |
|  |  |  | glutaredoxin-1 | 1.426 | * | heterogeneous nuclear ribonucleoprotein A1 isoform X1 | 1.207 | * |
|  |  |  | 5-hydroxyisourate hydrolase-like | 1.365 | * | endoplasmic reticulum resident protein 29 isoform X1 | 1.672 | * |
|  |  |  | heterogeneous nuclear ribonucleoprotein D0 | 1.338 | * | histidine triad nucleotide-binding protein 2, mitochondrial | 1.243 | * |
|  |  |  | fibronectin isoform X1 | 1.896 | * | short-chain specific acyl-CoA dehydrogenase, mitochondrial | 1.342 | * |
|  |  |  | inosine-5~-monophosphate dehydrogenase 2 isoform X1 | 1.352 | * | complement C3 | 1.959 | * |
|  |  |  | heterogeneous nuclear ribonucleoprotein L isoform X1 | 1.295 | * | ribonuclease inhibitor | 1.218 | * |
|  |  |  | platelet-activating factor acetylhydrolase IB subunit beta | 1.465 | * | galectin-1 | 1.244 | * |
|  |  |  | tenascin-X | 1.44 | * | acyl-CoA-binding protein isoform X1 | 1.213 | * |
|  |  |  | voltage-dependent L-type calcium channel subunit beta-1 isoform X3 | 1.246 | * | COP9 signalosome complex subunit 1 | 1.753 | * |
|  |  |  | heterogeneous nuclear ribonucleoprotein M isoform X1 | 1.272 | * | proactivator polypeptide isoform X1 | 1.226 | * |
|  |  |  | transcription factor A, mitochondrial | 1.254 | * | periostin isoform X1 | 2.732 | * |
|  |  |  | proteasome subunit alpha type-4 | 1.289 | * | D-dopachrome decarboxylase-like | 1.482 | * |
|  |  |  | kynurenine--oxoglutarate transaminase 1 | 1.359 | * | tubulin beta-2A chain isoform X1 | 1.687 | * |
|  |  |  | glutathione S-transferase omega-1 isoform X1 | 1.396 | * | prolyl endopeptidase | 1.25 | * |
|  |  |  | NADP-dependent malic enzyme, mitochondrial | 1.652 | * | 60S ribosomal protein L26 | 1.225 | * |
|  |  |  | calpain small subunit 1 | 1.301 | * | protein S100-A13 | 1.201 | * |
|  |  |  | protein FAM98B | 1.424 | * | ubiquitin carboxyl-terminal hydrolase isozyme L3 | 1.552 | * |
|  |  |  | glycerol-3-phosphate dehydrogenase | 1.206 | * | glutathione peroxidase 3 | 1.76 | * |
|  |  |  | plasminogen-like isoform X1 | 2.021 | * | mammalian ependymin-related protein 1 | 1.784 | * |
|  |  |  | cartilage intermediate layer protein 2 | 2.358 | * | collectin-10 | 8.835 | * |
|  |  |  | hemoglobin subunit zeta | 2.839 | * | 40S ribosomal protein S15a | 1.223 | * |
|  |  |  | haptoglobin | 1.817 | * | complement C4-A isoform X1 | 1.555 | * |
|  |  |  | leucine-rich repeat-containing protein 47 | 1.318 | * | mitogen-activated protein kinase 14 isoform X1 | 1.224 | * |
|  |  |  | ubiquitin carboxyl-terminal hydrolase 5 isoform X1 | 1.221 | * | peptidyl-prolyl cis-trans isomerase FKBP1A-like | 1.323 | * |
|  |  |  | nucleoside diphosphate kinase A isoform X1 | 1.296 | * | GMP reductase 1 | 1.56 | * |
|  |  |  | elongation factor 1-delta isoform X4 | 1.476 | * | peptidyl-prolyl cis-trans isomerase A-like | 1.327 | * |
|  |  |  | prefoldin subunit 1 | 1.289 | * | fibrinogen beta chain isoform X1 | 3.644 | * |
|  |  |  | acylamino-acid-releasing enzyme | 1.567 | * | gelsolin isoform X5 | 1.492 | * |
|  |  |  | heterogeneous nuclear ribonucleoprotein A/B isoform X1 | 1.34 | * |  |  |  |
|  |  |  | heterogeneous nuclear ribonucleoprotein A1 isoform X1 | 1.514 | * |  |  |  |
|  |  |  | endoplasmic reticulum resident protein 29 isoform X1 | 1.579 | * |  |  |  |
|  |  |  | short-chain specific acyl-CoA dehydrogenase, mitochondrial | 1.35 | * |  |  |  |
|  |  |  | heterogeneous nuclear ribonucleoprotein H | 1.833 | * |  |  |  |
|  |  |  | 14-3-3 protein zeta/delta-like | 1.386 | * |  |  |  |
|  |  |  | complement C3 | 1.933 | * |  |  |  |
|  |  |  | alpha-actinin-4 isoform X1 | 1.228 | * |  |  |  |
|  |  |  | beta-adducin isoform X1 | 1.759 | * |  |  |  |
|  |  |  | galectin-1 | 1.607 | * |  |  |  |
|  |  |  | acyl-CoA-binding protein isoform X1 | 1.339 | * |  |  |  |
|  |  |  | serine/arginine-rich splicing factor 1 | 1.536 | * |  |  |  |
|  |  |  | nucleosome assembly protein 1-like 4 | 1.404 | * |  |  |  |
|  |  |  | bifunctional purine biosynthesis protein PURH | 1.277 | * |  |  |  |
|  |  |  | COP9 signalosome complex subunit 1 | 1.697 | * |  |  |  |
|  |  |  | D-dopachrome decarboxylase-like | 2.018 | * |  |  |  |
|  |  |  | non-specific lipid-transfer protein-like isoform X1 | 1.292 | * |  |  |  |
|  |  |  | tubulin beta-2A chain isoform X1 | 1.637 | * |  |  |  |
|  |  |  | glycine cleavage system H protein, mitochondrial | 1.316 | * |  |  |  |
|  |  |  | small nuclear ribonucleoprotein Sm D1 | 1.316 | * |  |  |  |
|  |  |  | prolyl endopeptidase | 1.385 | * |  |  |  |
|  |  |  | histidine triad nucleotide-binding protein 1 | 1.32 | * |  |  |  |
|  |  |  | cAMP-dependent protein kinase type II-alpha regulatory subunit isoform X1 | 1.35 | * |  |  |  |
|  |  |  | protein S100-A13 | 1.442 | * |  |  |  |
|  |  |  | ubiquitin carboxyl-terminal hydrolase isozyme L3 | 1.591 | * |  |  |  |
|  |  |  | glutathione peroxidase 3 | 1.817 | * |  |  |  |
|  |  |  | tubulin polymerization-promoting protein family member 3 | 1.481 | * |  |  |  |
|  |  |  | exportin-2 isoform X1 | 1.301 | * |  |  |  |
|  |  |  | peptidyl-prolyl cis-trans isomerase A-like | 1.419 | * |  |  |  |
|  |  |  | fibrinogen beta chain isoform X1 | 2.168 | * |  |  |  |
|  |  |  | gelsolin isoform X5 | 1.391 | * |  |  |  |
|  |  |  | ubiquitin fusion degradation protein 1 homolog isoform X1 | 1.419 | * |  |  |  |
| **Down-regulated** | | | | | | | | |
| 40S ribosomal protein S6 | 0.802 | * | vesicle-associated membrane protein-associated protein B/C isoform X1 | 0.734 | * | cytochrome c oxidase subunit 4 isoform 1, mitochondrial-like isoform X2 | 0.811 | * |
| caveolin-1 isoform X1 | 0.746 | * | cytochrome c1, heme protein, mitochondrial-like isoform X1 | 0.636 | * | 28S ribosomal protein S25, mitochondrial | 0.588 | * |
| CDGSH iron-sulfur domain-containing protein 1 | 0.702 | * | reticulon-2 | 0.607 | * | dual specificity phosphatase DUPD1 isoform X1 | 0.425 | * |
| ras-related C3 botulinum toxin substrate 1 | 0.756 | * | CDGSH iron-sulfur domain-containing protein 1 | 0.601 | * | c-1-tetrahydrofolate synthase, cytoplasmic | 0.824 | * |
| 40S ribosomal protein S25 | 0.758 | * | ATP synthase subunit g, mitochondrial | 0.816 | * | kelch-like protein 40 | 0.768 | * |
| fibrinogen gamma chain | 0.599 | * | cytochrome c oxidase subunit 7A2, mitochondrial-like | 0.698 | * | cytochrome b-c1 complex subunit 8-like isoform X7 | 0.774 | * |
| glycogen phosphorylase, brain form isoform X1 | 0.819 | * | neuroblast differentiation-associated protein AHNAK-like | 0.648 | * | glutathione reductase, mitochondrial isoform X1 | 0.755 | * |
| phosphoglycerate mutase 1 | 0.577 | * | NADH dehydrogenase | 0.774 | * | cytochrome c1, heme protein, mitochondrial-like isoform X1 | 0.684 | * |
| ruvB-like 1 | 0.77 | * | ATPase inhibitor, mitochondrial | 0.68 | * | nebulin isoform X1 | 0.79 | * |
| anionic trypsin-2-like | 0.666 | * | NADH dehydrogenase | 0.562 | * | reticulon-2 | 0.796 | * |
| collagen alpha-2(I) chain isoform X1 | 0.685 | * | NADH dehydrogenase | 0.598 | * | myosin-2 | 0.482 | * |
| NADH dehydrogenase | 0.705 | * | actin, alpha cardiac muscle 1 | 0.823 | * | PDZ and LIM domain protein 3 isoform X3 | 0.517 | * |
| histone H2A.V-like | 0.742 | * | sodium/potassium-transporting ATPase subunit alpha-2 | 0.727 | * | calsequestrin-2 | 0.749 | * |
| collagen alpha-1(VI) chain | 0.786 | * | 60S ribosomal protein L10a | 0.74 | * | sodium/potassium-transporting ATPase subunit beta-1 | 0.757 | * |
| synaptophysin-like protein 2 | 0.771 | * | ryanodine receptor 1 | 0.743 | * | cytochrome c oxidase subunit 7A2, mitochondrial-like | 0.815 | * |
| transgelin isoform X2 | 0.784 | * | NADH-cytochrome b5 reductase 3-like | 0.81 | * | leiomodin-3 isoform X2 | 0.822 | * |
| probable D-lactate dehydrogenase, mitochondrial | 0.747 | * | citrate lyase subunit beta-like protein, mitochondrial | 0.758 | * | protein NDRG2 isoform X3 | 0.719 | * |
| actin-related protein 2 isoform X2 | 0.767 | * | NADH dehydrogenase | 0.722 | * | ADP/ATP translocase 3, partial | 0.685 | * |
| citrate lyase subunit beta-like protein, mitochondrial | 0.787 | * | xin actin-binding repeat-containing protein 2-like | 0.774 | * | PDZ and LIM domain protein 3 isoform X1 | 0.811 | * |
| 60S ribosomal protein L22 | 0.723 | * | short/branched chain specific acyl-CoA dehydrogenase, mitochondrial | 0.652 | * | NADH dehydrogenase | 0.648 | * |
| NADH dehydrogenase | 0.783 | * | NADH dehydrogenase | 0.749 | * | ATP synthase subunit delta, mitochondrial isoform X1 | 0.656 | * |
| serine/arginine-rich splicing factor 3 | 0.726 | * | ATP synthase subunit f, mitochondrial isoform X1 | 0.583 | * | probable C->U-editing enzyme APOBEC-2 | 0.769 | * |
| endonuclease G, mitochondrial | 0.71 | * | NADH dehydrogenase | 0.654 | * | mitochondrial 2-oxoglutarate/malate carrier protein isoform X1 | 0.754 | * |
| fibrillin-1 | 0.534 | * | tubulin-specific chaperone A | 0.626 | * | xin actin-binding repeat-containing protein 1 isoform X1 | 0.755 | * |
| glycylpeptide N-tetradecanoyltransferase 1 | 0.802 | * | NADH dehydrogenase | 0.686 | * | Pyruvate dehydrogenase (acetyl-transferring) | 0.392 | * |
| leucine-rich PPR motif-containing protein, mitochondrial | 0.8 | * | cytochrome c oxidase subunit 6C-like isoform X2 | 0.5 | * | Hemoglobin subunit beta | 0.833 | * |
| ras-related protein R-Ras | 0.673 | * | nebulin-related-anchoring protein | 0.747 | * | actin, alpha cardiac muscle 1 | 0.806 | * |
| NADH dehydrogenase | 0.788 | * | aspartyl/asparaginyl beta-hydroxylase isoform X2 | 0.712 | * | glutathione S-transferase Mu 2 | 0.57 | * |
| 40S ribosomal protein S7 | 0.735 | * | ATP synthase subunit b, mitochondrial | 0.765 | * | heat shock protein beta-7 isoform X1 | 0.751 | * |
| telethonin | 0.811 | * | striated muscle preferentially expressed protein kinase | 0.662 | * | sodium/potassium-transporting ATPase subunit alpha-2 | 0.738 | * |
| ubiquinone biosynthesis protein COQ9, mitochondrial | 0.811 | * | mitochondrial carnitine/acylcarnitine carrier protein | 0.615 | * | potassium voltage-gated channel subfamily V member 2 | 0.746 | * |
| delta-1-pyrroline-5-carboxylate dehydrogenase, mitochondrial | 0.805 | * | delta-1-pyrroline-5-carboxylate dehydrogenase, mitochondrial | 0.774 | * | cytochrome c oxidase subunit II (mitochondrion) | 0.765 | * |
| NADH-cytochrome b5 reductase 1-like | 0.648 | * | myosin-13 | 0.478 | * | synaptophysin-like protein 2 | 0.767 | * |
| glycogen | 0.759 | * | kelch-like protein 41 | 0.772 | * | dnaJ homolog subfamily A member 4 isoform X1 | 0.654 | * |
| myosin-3 | 0.587 | * | serine/threonine-protein kinase Nek7 isoform X2 | 0.69 | * | xin actin-binding repeat-containing protein 2-like | 0.699 | * |
| protein S100-A4 | 0.777 | * | supervillin | 0.789 | * | short/branched chain specific acyl-CoA dehydrogenase, mitochondrial | 0.622 | * |
| mast cell carboxypeptidase A isoform X1 | 0.385 | * | nitrilase homolog 1 isoform X1 | 0.677 | * | histone-lysine N-methyltransferase Smyd1 | 0.773 | * |
| collagen alpha-2(IV) chain | 0.648 | * | NADH-cytochrome b5 reductase 1-like | 0.568 | * | dnaJ homolog subfamily A member 2 | 0.728 | * |
| carnitine O-acetyltransferase | 0.83 | * | myosin-3 | 0.399 | * | PDZ and LIM domain protein 3 isoform X5 | 0.635 | * |
| poly(rC)-binding protein 2 isoform X1 | 0.732 | * | glycogen | 0.785 | * | tubulin-specific chaperone A | 0.517 | * |
| calcium/calmodulin-dependent protein kinase type II subunit delta | 0.691 | * | LETM1 and EF-hand domain-containing protein 1, mitochondrial | 0.713 | * | NADH dehydrogenase | 0.833 | * |
| up-regulated during skeletal muscle growth protein 5 | 0.638 | * | choline-phosphate cytidylyltransferase A | 0.759 | * | glutathione S-transferase Mu 2-like isoform X4 | 0.6 | * |
| ATP synthase protein 8 | 0.688 | * | mast cell carboxypeptidase A isoform X1 | 0.405 | * | Protein ADP-ribosylarginine | 0.769 | * |
| long-chain fatty acid transport protein 1 | 0.685 | * | electron transfer flavoprotein-ubiquinone oxidoreductase, mitochondrial isoform X1 | 0.749 | * | glycogenin-1 isoform X1 | 0.592 | * |
| histone H3.3 | 0.672 | * | carnitine O-acetyltransferase | 0.81 | * | cytochrome c oxidase subunit 6C-like isoform X2 | 0.704 | * |
| acetyl-coenzyme A synthetase 2-like, mitochondrial isoform X1 | 0.596 | * | dual specificity phosphatase DUPD1 isoform X1 | 0.515 | * | nebulin-related-anchoring protein | 0.567 | * |
| NADH dehydrogenase | 0.824 | * | transmembrane protein 143 | 0.539 | * | dnaJ homolog subfamily C member 11 | 0.561 | * |
| FH1/FH2 domain-containing protein 1 | 0.77 | * | cytoplasmic dynein 1 heavy chain 1 | 0.807 | * | aspartyl/asparaginyl beta-hydroxylase isoform X2 | 0.644 | * |
| chaperone activity of bc1 complex-like, mitochondrial isoform X3 | 0.774 | * | apolipoprotein O | 0.822 | * | long-chain-fatty-acid--CoA ligase 1 isoform X2 | 0.803 | * |
| NADH dehydrogenase | 0.817 | * | NADH dehydrogenase | 0.593 | * | carnitine O-palmitoyltransferase 1, muscle isoform isoform X3 | 0.752 | * |
| phosphorylase b kinase regulatory subunit alpha, skeletal muscle isoform isoform X1 | 0.816 | * | up-regulated during skeletal muscle growth protein 5 | 0.557 | * | O-acetyl-ADP-ribose deacetylase MACROD1 | 0.758 | * |
| mitochondrial 2-oxoglutarate/malate carrier protein isoform X1 | 0.731 | * | long-chain fatty acid transport protein 1 | 0.635 | * | myosin-binding protein C, slow-type isoform X1 | 0.654 | * |
| hemoglobin subunit zeta | 0.766 | * | ATP synthase protein 8 | 0.517 | * | supervillin | 0.814 | * |
| unconventional myosin-Ic | 0.792 | * | AMP deaminase 1 isoform X1 | 0.833 | * | phosphate carrier protein, mitochondrial isoform X2 | 0.7 | * |
| proteasome-associated protein ECM29 homolog | 0.595 | * | NADH dehydrogenase | 0.817 | * | LIM and cysteine-rich domains protein 1 isoform X1 | 0.731 | * |
| NADH dehydrogenase | 0.689 | * | platelet glycoprotein 4 | 0.739 | * | myosin-3 | 0.758 | * |
| fibrinogen alpha chain | 0.546 | * | protein NDRG2 isoform X3 | 0.796 | * | cytochrome c oxidase subunit 7A1, mitochondrial-like | 0.75 | * |
| ferritin heavy chain | 0.469 | * | NADH dehydrogenase | 0.766 | * |  |  |  |
| 2-methoxy-6-polyprenyl-1,4-benzoquinol methylase, mitochondrial | 0.783 | * | ADP/ATP translocase 3, partial | 0.663 | * |  |  |  |
| glutamic-oxaloacetic transaminase 2, aspartate aminotransferase 2, mitochondrial | 0.83 | * | uncharacterized protein LOC101957476 | 0.789 | * |  |  |  |
| ADP/ATP translocase 1 | 0.759 | * | NADH dehydrogenase | 0.704 | * |  |  |  |
| periostin isoform X1 | 0.377 | * | mitochondrial 2-oxoglutarate/malate carrier protein isoform X1 | 0.617 | * |  |  |  |
| collagen alpha-1(I) chain | 0.426 | * | ATP synthase subunit delta, mitochondrial isoform X1 | 0.77 | * |  |  |  |
| long-chain-fatty-acid--CoA ligase 1 isoform X2 | 0.77 | * | Pyruvate dehydrogenase (acetyl-transferring) | 0.393 | * |  |  |  |
| collectin-10 | 0.193 | * | mitochondrial carrier homolog 2 | 0.588 | * |  |  |  |
| O-acetyl-ADP-ribose deacetylase MACROD1 | 0.79 | * | protein-cysteine N-palmitoyltransferase HHAT-like protein | 0.738 | * |  |  |  |
| carnitine O-palmitoyltransferase 1, muscle isoform isoform X3 | 0.811 | * | D-beta-hydroxybutyrate dehydrogenase, mitochondrial | 0.785 | * |  |  |  |
| tropomodulin-1 | 0.701 | * | glutathione S-transferase Mu 2 | 0.665 | * |  |  |  |
| cullin-5 | 0.774 | * | potassium voltage-gated channel subfamily V member 2 | 0.603 | * |  |  |  |
| fibrinogen beta chain isoform X1 | 0.602 | * | proteasome-associated protein ECM29 homolog | 0.685 | * |  |  |  |
| coenzyme Q-binding protein COQ10 homolog A, mitochondrial isoform X1 | 0.755 | * | cytochrome c oxidase subunit II (mitochondrion) | 0.623 | * |  |  |  |
| cytochrome c oxidase subunit 7A1, mitochondrial-like | 0.664 | * | NADH dehydrogenase | 0.513 | * |  |  |  |
| prohibitin-2 isoform X1 | 0.829 | * | histone-lysine N-methyltransferase Smyd1 | 0.653 | * |  |  |  |
| perilipin-1 | 0.733 | * | ADP/ATP translocase 1 | 0.553 | * |  |  |  |
|  |  |  | calcium-binding mitochondrial carrier protein Aralar1 isoform X1 | 0.786 | * |  |  |  |
|  |  |  | dystrophin-like, partial | 0.765 | * |  |  |  |
|  |  |  | obscurin, partial | 0.829 | * |  |  |  |
|  |  |  | glycogenin-1 isoform X1 | 0.672 | * |  |  |  |
|  |  |  | flotillin-1 | 0.806 | * |  |  |  |
|  |  |  | filamin-B | 0.743 | * |  |  |  |
|  |  |  | long-chain-fatty-acid--CoA ligase 1 isoform X2 | 0.617 | * |  |  |  |
|  |  |  | O-acetyl-ADP-ribose deacetylase MACROD1 | 0.616 | * |  |  |  |
|  |  |  | carnitine O-palmitoyltransferase 1, muscle isoform isoform X3 | 0.564 | * |  |  |  |
|  |  |  | NADH dehydrogenase | 0.561 | * |  |  |  |
|  |  |  | phosphate carrier protein, mitochondrial isoform X2 | 0.457 | * |  |  |  |
